# Supplementary material for: A randomized trial of Trendelenburg position for acute moderate ischemic stroke
Source: Nat Commun. 2023 May 5;14:2592. doi: 10.1038/s41467-023-38313-y (PMC10163013; doi:10.1038/s41467-023-38313-y)
Supplement: Supplementary file 1 — Supplementary Information [file 41467_2023_38313_MOESM1_ESM.pdf]

Supplementary Information for “A randomized trial of Trendelenburg position for acute moderate ischemic stroke”

Supplementary Methods

Definition of early neurological deterioration: early neurological deterioration is defined as an increase of four or more NIHSS compared to baseline after stroke within 48 hours.[1]

Definition of stroke: stroke was defined as an acute focal central neurological deficit lasting >24 hours that resulted in irreversible brain damage or body impairment by a vascular cause.[2]

Definition of other vascular events: other vascular events include pulmonary embolism, peripheral vessel incident, and cardiovascular incident.

Definition of intracranial hemorrhage: Intracranial hemorrhage was defined according to the ECASS-1 study.[3]

Clinicaltrials.gov registration

The HOPES2 trial is a prospective, random, open-label, blinded endpoint and multi-center study, which is registered at clinicaltrials.gov on 16th Nov 2018 (NCT03744533). The trial was initially set-up on 26th Nov 2018 and recruited their first patient on 4th Dec 2018.

1. National Institute of Neurological Disorders and Stroke rt-PA Stroke Study Group. Tissue plasminogen activator for acute ischemic stroke. *N Engl J Med*. 1995; **333:**1581-7.
2. Campbell BCV, Khatri P. Stroke. *Lancet*. 2020; **396:** 129-42.
3. Hacke W, Kaste M, Fieschi C, et al. Intravenous thrombolysis with recombinant tissue plasminogen activator for acute hemispheric stroke. The European Cooperative Acute Stroke Study (ECASS). *JAMA*. 1995; **274:** 1017-25.

Supplementary Table 1. Baseline characteristics and procedural details for the per-protocol population.

	HDP group (*n*=42)	Control group (*n*=47)	*P* value
Age, years	61.1 (11.3)	64.2 (11.8)	0.201
Sex			0.924
Male	30 (71.4%)	34 (72.3%)	
Female	12 (28.6%)	13 (27.7%)	
Risk factors			
Hypertension	24/41 (58.5%)	29/46 (63.0%)	0.667
Diabetes	12/41 (29.3%)	11/46 (23.9%)	0.572
Hyperlipidemia	3 (7.1%)	2/46 (4.3%)	0.572
Coronary heart disease	7 (16.7%)	3/46 (6.5%)	0.134
Previous stroke	14 (33.3%)	16 (34.0%)	0.944
Current smoker	24 (57.1%)	24 (51.1%)	0.566
Current drinker	23 (54.8%)	20 (42.6%)	0.250
Blood pressure at randomization, mmHg			
Systolic	160.8 (26.2)	159.9 (24.7)	0.870
Diastolic	88.1 (13.4)	88.2 (11.6)	0.983
Blood pressure at 24 hours, mmHg			
Systolic	131.6 (62.2)	129.6 (60.4)	0.880
Diastolic	94.3 (30.2)	98.9 (31.6)	0.512
Blood pressure at 7 days, mmHg			
Systolic	132.9 (30.5)	132.0 (26.6)	0.896
Diastolic	94.6 (29.2)	87.5 (23.5)	0.286
NIHSS score at randomization[a]	9 (7-11)	9 (6-11)	0.702
Medication during HDP			0.946
Mono antiplatelet	17 (40.5%)	19/46 (41.3%)	
Dual antiplatelet	15 (35.7%)	15/46 (32.6%)	
Antiplatelet + anticoagulant	10 (23.8%)	12/46 (26.1%)	

Lipid-lowering therapy			0.208
high intensity	28/38 (73.7%)	26/43 (60.5%)	
non high intensity	10/38 (26.3%)	17/43 (39.5%)	
Responsible vessels			0.264
Extracranial ICA	6/39 (15.4%)	4/45 (8.9%)	
Intracranial ICA	8/39 (20.5%)	5/45 (11.1%)	
M1 segment of MCA	25/39 (64.1%)	36/45 (80.0%)	
The degree of responsible vessel stenosis			0.941
Moderate (50-69%)	8/39 (20.5%)	9/44 (20.5%)	
Severe (70-99%)	11/39 (28.2%)	11/44 (25.0%)	
Occlusion	20/39 (51.3%)	24/44 (54.5%)	
Onset to randomization time (h)	15.5 (6.8-21.3)	10.0 (7.0-19.0)	0.246
ICU care	10 (23.8%)	8 (17.0%)	0.426

Data are No.(%) or No./total (%), mean (SD), or median (IQR). HDP = head-down position. ICA = internal carotid artery. MCA = middle cerebral artery. HDP = head down position. NIHSS = National Institute of Health Stroke Scale. Baseline characteristics were compared with Student's t test if normally distributed or Mann-Whitney test if not normally distributed for continuous variables, and χ^2 for categorical variables. All tests were two-tailed. [a]Scores range from 0 to 42, with higher scores indicating more severe neurological deficit.

Supplementary Table 2. Primary and secondary outcomes in the per-protocol population.

	HDP group (*n*=42)	Control group (*n*=47)	Unadjusted		Adjusted[a]	
			OR (95%CI)	*P* value	OR (95%CI)	*P* value
**Primary outcome**						
mRS score 0-2 at 90 days	29 (69.0%)	24 (51.1%)	2.14(0.90-5.10)	0.087	2.76 (0.96-7.96)	0.061
**Secondary outcomes**						
mRS score 0-1 at 90 days	20 (47.6%)	12 (25.5%)	2.65 (1.09-6.47)	0.032*	2.80 (1.00-7.85)	0.050*
Improvement in mRS according to category at day 90[b]			2.69(1.26-5.76)	0.011*	3.31 (1.47-7.42)	0.004*
0	10 (23.8%)	3 (6.4%)				
1	10 (23.8%)	9 (19.1%)				
2	9 (21.4%)	12 (25.5%)				
3	9 (21.4%)	11 (23.4%)				
4	1 (2.4%)	6 (12.8%)				
5	2 (4.8%)	1 (2.1%)				
6	1 (2.4%)	5 (10.6%)				
Early neurological deterioration within 48 hours[c]	0	2 (4.3%)				

Change in NIHSS score at day 12 from baseline[d]	3.3 (3.4)	0.6 (7.0)	-0.15(-0.26- -0.05)	0.005*	-0.17(-0.27- -0.06)	0.002*
Death within 90 days	1 (2.4%)	5 (10.6%)	0.21 (0.02-1.83)	0.156	0.26 (0.02-3.67)	0.318

Frequency data are No.(%), or mean (SD). HDP = head-down position. mRS = modified Rankin scale. NIHSS = National Institute of Health Stroke Scale. Treatment effect is presented as odds ratio (95% CI) of HDP group versus control group, analyzed by unadjusted and adjusted binary logistic regression. [a] Adjusted for key prognostic covariates (age, NIHSS score at randomization, the degree of responsible vessel stenosis, responsible vessels and onset to randomization time). [b] The outcome was assessment of scores across all seven levels of the mRS (ranging from 0 [no symptoms] to 6 [death]), done using a shift analysis of the ordinal data. [c] Early neurological deterioration was defined as ≥ 4 increase in NIHSS score within 48 hours, but not result of cerebral haemorrhage. [d] NIHSS scores range from 0 to 42, with higher scores indicating greater stroke severity. Log(NIHSS+1) was analyzed using generalized linear model. Treatment effect is presented as geometric mean ratio. All tests were two-tailed. No adjustments were made for multiple comparisons. $*P < 0.05$. Details be provided as the method (in-person vs telephone) and source (patient vs surrogate) of the mRS outcomes.

Supplementary Table 3. Sensitive Analysis for Missing Primary Outcome in Dropout Subjects in the modified intention-to-treat population.

	Without primary outcome imputation				With primary outcome imputation				
	HDP group (n=42)	Control group (n=47)	Odds ratio (95% CI)	*P* value	HDP group (n=46)	Control group (n=48)	Odds ratio (95% CI)	*P* value	Imputation methods
mRS score 0-2 within 90 days, No.(%)	29 (69.0%)	24 (51.1%)	2.14 (0.90-5.10)	0.087	30 (65.2%)	24 (50.0%)	1.88 (0.82-4.30)	0.138	Last observation carried forward
					30 (65.2%)	24 (50.0%)	1.88 (0.82-4.30)	0.138	Worst-case scenario
					32 (69.6%)	25 (52.1%)	2.10 (0.90-4.90)	0.085	Best-case scenario

Frequency data are No.(%). HDP = head-down position. mRS = modified Rankin scale. Treatment effect is presented as odds ratio (95% CI) of HDP group versus control group, analyzed by binary logistic regression.

Supplementary Table 4. Laboratory examination in the per-protocol population

Laboratory items	HDP group (n=42)	Control group (n=47)	*p* value
White blood cells, 10^9/L	8.1 (2.3)	7.4 (2.0)	0·196
Central granulocyte, 10^9/L	6.0 (2.3)	5.4 (1.9)	0·198
Lymphocyte cell, 10^9/L	1.6 (0.7)	1.7 (1.1)	0·626
Red blood cells, 10^12/L	4.6 (0.5)	4.5 (0.5)	0·254
Hemoglobin, g/L	141.5.(17.4)	137.5 (17.8)	0·320
Blood platelet, 10^9/L	226.2 (47.6)	219.2 (52.8)	0·540
Total protein, g/L	65.0 (5.7)	63.8 (9.1)	0.495
Serum albumin assay, g/L	41.5 (5.1)	41.2 (5.6)	0·804
Globin, g/L	24.1 (4.1)	24.6 (4.3)	0·596
Blood urea nitrogen, mmol/L	5.6 (1.9)	5.6 (1.8)	0.964
Serum creatinine, μmol/L	83.4 (62.0)	65.8 (18.2)	0.084
Cystatin C	0.9 (0.4)	0.9 (0.2)	0.796
Serum uric acid, μmol/L	293.0 (106.4)	297.7 (108.9)	0.849
Homocysteine, μmol/L	14.5 (8.0)	16.0(11.3)	0.542
Serum triglyceride, mmol/L	1.6 (1.2)	1.3 (0.4)	0.063
Serum total cholesterol, mmol/L	5.0 (1.2)	4.6 (1.1)	0.172
Low density lipoprotein, mmol/L	3.0 (1.0)	2.7 (0.9)	0·322
High density lipoprotein, mmol/L	1.1 (0.3)	1.2 (0.3)	0·363
Glycohemoglobin	6.6 (1.9)	6.2 (1.3)	0.372
Urine specific gravity	1.0 (0.1)	1.0 (0.1)	0.200
C-reactive protein, mg/L *	10.2 (19.0)	6.6 (13.3)	0.451
High sensitive C-reactive protein, mg/L †	9.0 (13.3)	15.7 (19.6)	0.288
B-type Natriuretic Peptide, pg/mL ‡	206.8 (253.7)	278.8 (355.6)	0.408

Data are n/N (%), mean (SD), or median (IQR). HDP = head down position. * Data on C-reactive protein available for 48 patients. † Data on high sensitive C-reactive protein available for 31 patients. ‡ Data on B-type Natriuretic Peptide available for 51 patients.

Supplementary Note 1: Original protocol (v 1.0)

HOPES2 Protocol

Head dOwn-Position for acutE moderate ischemic Stroke with large artery atherosclerosis: a prospective, random, multi-center trial

Chief investigator: Prof. Hui-Sheng Chen

Department of Neurology,

General Hospital of Shenyang Military Region

83 Wen Hua Road, Shen Yang 110840, P.R. China

Tel: 86-24-28897511

E-mail: chszh@aliyun.com

Sponsor: General Hospital of Shenyang Military Region

Ethics committee of General Hospital of Shenyang Military Region approval no. k (2018) 38

Protocol version and date: Version 1.0, 20th Nov 2018

Abstract

Rationale: To date, there is no effective neuroprotection for acute ischemic stroke (AIS), except reperfusion strategy such as intravenous thrombolysis and mechanical thrombectomy. Clinical studies suggested that lying-flat position may increase cerebral blood flow. However, there is lack of convincing evidence for the neuroprotective effect of head position on ischemic stroke.

Aim: To explore the efficacy and safety of head-down position (HDP) for acute moderate ischemic stroke.

Methods and design: In this prospective, multi-center, open-label, blind-endpoint, randomized, control trial, eligible patients with moderate ischemic stroke were randomly assigned (1:1) into HDP group receiving Trendelenburg (-20 degree) as an adjunct to guideline-based treatment, and control group only receiving guideline-based treatment.

Study outcome: The primary outcome is excellent functional outcome, defined as modified Rankin Scale 0-1 at 90 days.

Keywords

Head-down position, acute ischemic stroke, protocol

Introduction

Currently, the guideline recommended reperfusion treatments such as intravenous thrombolysis and mechanical thrombectomy as the most effective treatment for acute ischemic stroke[1]. However, these treatments are limited by a strict time window and technique requirements, which obviously decreased eligible patients for reperfusion treatments.

Good collateral circulation has been demonstrated to provide compensatory blood supply to rescue the ischemic penumbra and reduce the infarct volume[2], which in turn improves the prognosis. How to effectively and safely improve collateral circulation remains a significant clinical challenge. The effect of head position on stroke has been investigated[3]. It is generally accepted that lying-flat position may increase blood flow and improved oxygenation[4]. The Head Positioning in Acute Stroke Trial (HeadPoST) is the first big sample study to compare the effects of the lying-flat versus sitting-up position in AIS patients[5], and the results showed that the lying-flat position was safe, but ineffective. The negative results may be due to the broad inclusion of stroke patients, and we argue that the patients with large artery atherosclerosis (LAA) etiology should be benefited from head position intervention.

In theory, compared with lying-flat position, the aggressive head-down position (i.e., fully supine Trendelenburg[6]) would more significantly increase blood flow to the ischemic penumbra and improve oxygenation of brain in the first hours or days after stroke[7]. Our recent results of animal and preliminary clinical studies showed that head-down position (HDP) may significantly improve neurological function.

Given that HDP is simple and clinically easy to operate, and may increase brain perfusion and improve collateral circulation in theory, we designed the prospective, multi-center, open-label, blinded-endpoint, randomized control study, aiming to explore the efficacy and safety of HDP for acute moderate anterior circulation stroke patients with large artery atherosclerosis within 24 hours of onset.

Methods

Design

HOPES2 is a prospective, multi-center, open-label, blind-endpoint, randomized control study to assess the efficacy and safety of HDP in moderate anterior circulation stroke patients with large artery atherosclerosis (LAA). The main purpose of this study is to test the hypothesis that two weeks' HDP therapy within 24 hours from symptom onset exerts neuroprotective effect on AIS patients with moderate deficit and LAA etiology.

Intervention

In this trial, eligible patients will be randomly assigned (1:1) using a computer-generated randomization sequence with block size of four and sealed envelopes, prepared by an independent statistician, into either HDP group receiving Trendelenburg (-20 degree) as an adjunct to guideline-based treatment, or control group only receiving guideline-based treatment (Figure 1). In the HDP group, the subjects are lowered to -20 degree in a supine position between 8:00-22:00 within 24h post-randomization as long as possible. During the procedure, when subjects feel intolerable, the head position will be elevated to lying flat (0 degree) for 5-10 minutes, and then the above procedure is repeated. After 24h post-randomization, the head-down procedures with -20 degree in a supine position with duration of 1-1.5 hour will be performed three times daily at 9:00-11:00, 15:00-17:00 and 20:00-22:00, respectively. In the control group, patients will be treated according to AHA/ASA guidelines for early management of ischemic stroke without any intervention of head position[1].

Figure 1. Study schema

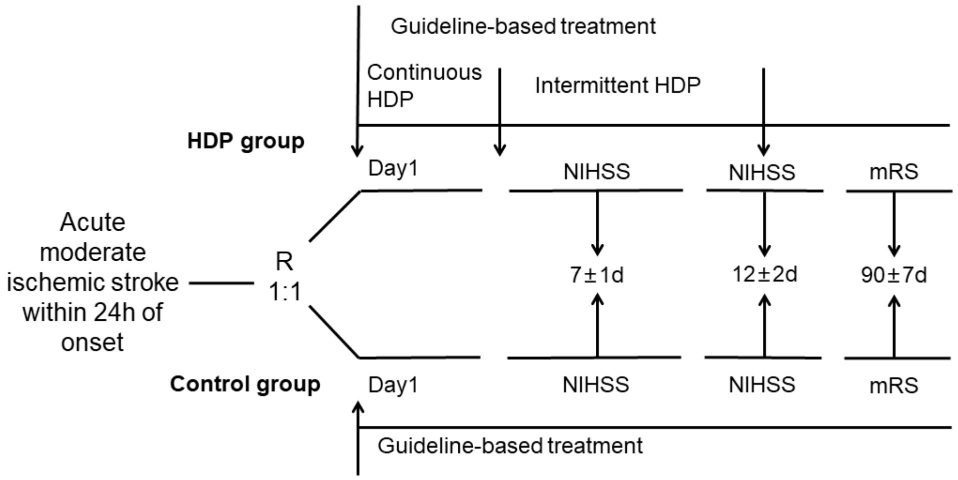

HDP: head-down position. mRS: modified Rankin Scale. NIHSS: National Institutes of Health Stroke Scale.

Patient population

A total of 100 patients with acute moderate ischemic stroke in ten centers are expected in China between December 2018 and December 2021. There are 50 subjects in the experimental group and control group, respectively. The detailed inclusion/exclusion criteria are listed in Table 1.

Table 1. The inclusion/exclusion criteria

**Inclusion criteria:**	
1)	Patient age ≥ 18 years.
2)	Acute ischemic stroke confirmed by CT or MRI
3)	The time from onset to treatment: within 24 h
4)	Moderate neurological deficit: 6 ≤ National Institute of Health stroke scale (NIHSS) ≤ 22.
5)	Large artery atherosclerosis etiology based on the Trial of Org 10172 in Acute Stroke Treatment (TOAST) criteria (culprit artery stenosis ≥ 50%, confirmed by CTA or MRA).
6)	Evidence of unilateral large-vessel stenosis or occlusion of the internal carotid artery or middle cerebral artery (MCA) M1 or proximal M2 segment.

7)	first stroke onset or past stroke without obvious neurological deficit (mRS≤1)
8)	Signed informed consent from the patients, or their legally authorized representative.
**Exclusion criteria:**	
1)	Patients with disturbance of consciousness.
2)	Patients who plan to undergo or have completed thrombolysis or mechanical thrombectomy.
3)	Hemorrhagic stroke or combined stroke.
4)	Complicated with serious diseases, such as liver and kidney insufficiency, malignant tumor, etc.
5)	A history of stroke with severe sequelae (mRS ≥ 2).
6)	Other etiologies, such as cardiogenic embolism, arteritis, arterial dissection, moyamoya disease, etc.
7)	Previous history of intracerebral hemorrhage within 1 year.
8)	Any contraindication to head-down position (e.g. active vomiting, pneumonia, uncontrolled heart failure).
9)	Planned carotid or intracranial revascularization within 3 months.
10)	Enrolled in other clinical trials within 3 months.
11)	Pregnant or lactating women.
12)	Any inappropriate patient assessed by the researcher.

mRS: modified Rankin Scale; NIHSS: National Institutes of Health Stroke Scale.

Standard protocol approvals, registrations, and patient consents

The trial was registered on clinicaltrial.gov (NCT03744533). The protocol and data collection of the trial have been approved by the ethics committee of General Hospital of Shenyang Military Region and all participating sites. All subjects or their representatives will provide written informed consents before inclusion into the trial.

Data collection and follow-up

Demography and clinically relevant data are recorded at the time of entry into the study. All enrolled patients have face-to-face or telephone interview at baseline, 7 days, 12 days, and 90 days post-randomization, respectively. Data are recorded, identified, and assessed by experienced neurologists blinded to the intervention. Baseline and follow-up NIHSS scores were evaluated by the same neurologist who were not blinded to treatment allocation. Final 90-day mRS was evaluated by one qualified personnel who were blinded to treatment allocation according to a standardized procedure manual in each study center. To ensure validity and reproducibility of the evaluation, we held a training course for all investigators at each center. Concomitant medications and adverse events within 90 days after randomization will be recorded in detail by investigators.

Outcomes

The primary endpoint is the proportion of modified Rankin Scale (mRS) 0-1 at 90 days. The secondary endpoints are as follows: (1) proportion of mRS 0-2 at 90 days; (2) distribution of mRS score at 90 days; (3) proportion of early neurological deterioration (END), defined as more than 4 increase in National Institute of Health stroke scale (NIHSS) within 48 hours, but not result of cerebral hemorrhage; (4) change in NIHSS score compared with baseline at 12 days; (5) occurrence of stroke or other vascular events at 90 days; (6) proportion of death due to any cause within 90 days.

Quality control

Before the beginning of the study, all the investigators at each center attended training sessions to review the protocol and procedures. An independent Data Monitoring Committee (DMC) will perform to assure fidelity of conduct of the study according to the protocol and Good Clinical Practice (GCP). The primary purpose of the termination is to protect the rights and interests of the subjects and to avoid unnecessary economic losses. If the therapy shows a statistically significant difference of efficacy and/or safety over the other, the DMC has the right to terminate the study unconditionally.

Data management and monitoring

Data will be stored in the case report form (CRF). DMC is established to ensure ongoing monitoring of data security, such as hemorrhagic events and other adverse events, etc. Clinical outcome events (stroke, death, intracerebral hemorrhage, cardiopulmonary events, and stroke-associated pneumonia) will also be adjudicated by the independent DMC. Neuroimaging associated with clinical events will be read locally, reports will be included in adjudication packets, and actual images will be sent to the central reader from local investigators.

All AEs monitoring

All information about AEs should be recorded on the AEs page of the case report. All relevant SAEs are reviewed and adjudicated centrally in order to ensure that they meet the same diagnostic criteria.

Sample size determination

No formal sample size calculation was performed due to no relevant data from previous trial. For this exploratory trial, the sample size (50 patients per group) was determined primarily based on the suggestion of the Steer Committee.

Statistical analysis

All efficacy analyses will be performed according to the intention-to-treat principle, which comprises patients who received the allocated treatment and completed the assessment period. Baseline characteristics and procedural details will be compared with Student's t test if normally distributed or Wilcoxon test if non-normally distributed. Treatment effect will be presented as odds ratio (95% CI) of HDP group versus control group, analyzed by binary logistic regression. Shift analysis of the mRS scores at 90 days will be performed using ordinal logistic regression. Change in NIHSS score at 12 days from baseline will be calculated with generalized estimating equation. A sensitivity analysis will be undertaken for the key outcomes adjusted for confounding

covariates (age; NIHSS score at randomization; and the degree of vascular stenosis). Descriptive statistics of proportions will be used for the safety data. Continuous data are presented as mean (SD) or median (IQR) as appropriate. For categorical variables, absolute and relative frequencies are presented. there is statistical significance if *P* value < 0.05. Analyses will be performed with statistical software of IBM SPSS Statistics 24.

Study organization and funding

The protocol was designed by Hui-Sheng Chen and discussed by the academic team. Steering Committee is made up of external scientific advisors, and will monitor the research and data regularly. The study is supported by grants from the National Natural Science Foundation of the Peoples Republic of China (8207147) and the Science and Technology Project Plan of Liao Ning Province (2018225023, 2019JH2/10300027).

Discussion

It is recommended by guidelines that reperfusion therapies such as intravenous thrombolysis and mechanical thrombectomy can provide compensatory blood supply to save the ischemic penumbra and reduce the infarct volume, which improves the prognosis for acute ischemic stroke.[1] Up to date, there is a lack of effective method to improve cerebral perfusion and collateral circulation except reperfusion treatments.

The effect of head position on stroke has been investigated. For example, head position has an impact on cerebral blood flow, cerebral perfusion, and intracranial pressure.[8-10] The HeadPoST found that lying-flat position did not improve neurological function, compared with the sitting-up position in AIS patients.[5]

The current study is the first clinical trial to investigate the effect of HDP (-20 degree) on functional outcome in AIS patients, which is quite different from these previous studies. Firstly, head-down position with-20 degree was adopted in this study. In theory, compared with lying-flat position, the aggressive HDP would more significantly increase blood flow in the ischemic penumbra and improve oxygenation of brain in the initial hours or days after stroke onset.[11] The intervention strategy was further supported by our recent unpublished data that HDP with -20 degrees and one hour duration after

ischemia-reperfusion can improve neurological function and reduce infarct volume in rats with middle cerebral artery occlusion model, and reversed early neurological deterioration and improved clinical outcomes in several cases with LAA. Secondly, the current study will enroll acute moderate ischemic stroke patients with LAA, while the HeadPoST study did not classify the severity of cerebral infarction and etiology. We argue that these patients should be most likely to benefit from neuroprotective therapy, because the neuroprotective effect will be underestimated in patients with mild neurological deficit, while the patients with severe neurological deficit who was mostly due to large artery occlusion would not be improved by neuroprotective treatment without the help of reperfusion treatment. In addition, the pathogenesis of LAA is mostly related to hypoperfusion, whose neurological function would be improved from increased cerebral perfusion due to head-down position. Thirdly, long head position intervention strategy will be used in the current study: constant HDP for 10 hours within 24 hours followed by HDP three times daily for 2 weeks, while only first 24 hours after randomization in HeadPoST study. We argue that long-term head position intervention may result in more benefit of neuroprotective effect.

In conclusion, we conduct a prospective, open label, blinded endpoint, multi-center, randomized control trial to explore the efficacy and safety of HDP with about two weeks' duration in acute moderate ischemic stroke patients with large artery atherosclerosis within 24 hours from onset.

References

1 Powers, W. J., Rabinstein, A. A., Ackerson, T., Aet al. 2018 Guidelines for the Early Management of Patients With Acute Ischemic Stroke: A Guideline for Healthcare Professionals From the American Heart Association/American Stroke Association. *Stroke* **49:** e46–e110.

2 Shuaib A, Butcher K, Mohammad AA, Saqqur M, Liebeskind DS. Collateral blood vessels in acute ischaemic stroke: a potential therapeutic target. *Lancet Neurol*.2011; **10:** 909-21.

3 Olavarría VV, Arima H, Anderson CS, et al. Head position and cerebral blood flow velocity in acute ischemic stroke: a systematic review and meta-analysis. *Cerebrovasc Dis* 2014; **37**: 401-8.

4 Olavarría VV, Lavados PM, Muñoz-Venturelli P, et al. Flat-head positioning increases cerebral blood flow

in anterior circulation acute ischemic stroke. A cluster randomized phase IIb trial. *Int J Stroke* 2018; **13**: 600-11.

5 Anderson CS, Arima H, Lavados P, et al. Cluster-Randomized, Crossover Trial of Head Positioning in Acute Stroke. *N Engl J Med* 2017; **376**: 2437-47.

6 Martin JT. The Trendelenburg position: a review of current slants about head down tilt. *AANA J* 1995; **63**: 29-36.

7 Ogawa Y, Yanagida R, Ueda K, Aoki K, Iwasaki K. The relationship between widespread changes in gravity and cerebral blood flow. *Environ Health Prev Med* 2016; **21**: 186-92.

8 Favilla CG, Mesquita RC, Mullen M, et al. Optical bedside monitoring of cerebral blood flow in acute ischemic stroke patients during head-of-bed manipulation. *Stroke* 2014; **45**: 1269-74.

9 Schwarz S, Georgiadis D, Aschoff A, Schwab S. Effects of body position on intracranial pressure and cerebral perfusion in patients with large hemispheric stroke. *Stroke* 2002; **33**: 497-501.

10 Lu SS, Ge S, Su CQ, et al. MRI of plaque characteristics and relationship with downstream perfusion and cerebral infarction in patients with symptomatic middle cerebral artery stenosis. *J Magn Reson Imaging* 2018; **48**: 66-73.

11 Ax M, Sanchez-Crespo A, SGE L, Mure M, Petersson J. The influence of gravity on regional lung blood flow in humans: SPECT in the upright and head-down posture. *J Appl Physiol (1985)* 2017; **122**: 1445-51.

12 Lee SJ, Lee DG. Distribution of atherosclerotic stenosis determining early neurologic deterioration in acute ischemic stroke. *PLoS One* 2017; **12:** e0185314.

13 Kim SJ, Seok JM, Bang OY, et al. MR mismatch profiles in patients with intracranial atherosclerotic stroke: a comprehensive approach comparing stroke subtypes. *J Cereb Blood Flow Metab* 2009; **29:** 1138-45.

Supplementary Note 2: Final protocol (v 2.0)

HOPES2 Protocol

Head dOwn-Position for acutE moderate ischemic Stroke with large artery atherosclerosis: a prospective, random, multi-center trial

Chief investigator: Prof. Hui-Sheng Chen

Department of Neurology,

General Hospital of Shenyang Military Region

83 Wen Hua Road, Shen Yang 110840, P.R. China

Tel: 86-24-28897511

E-mail: chszh@aliyun.com

Sponsor: General Hospital of Shenyang Military Region

Ethics committee of General Hospital of Shenyang Military Region approval no. k (2018) 38

Protocol version and date: Version 2.0, 19th Mar 2019

Abstract

Rationale: To date, there is no effective neuroprotection for acute ischemic stroke (AIS), except reperfusion strategy such as intravenous thrombolysis and mechanical thrombectomy. Clinical studies suggested that lying-flat position may increase cerebral blood flow. However, there is lack of convincing evidence for the neuroprotective effect of head position on ischemic stroke.

Aim: To explore the efficacy and safety of head-down position (HDP) for acute moderate ischemic stroke.

Methods and design: In this prospective, multi-center, open-label, blind-endpoint, randomized, control trial, eligible patients with moderate ischemic stroke were randomly assigned (1:1) into HDP group receiving Trendelenburg (-20 degree) as an adjunct to guideline-based treatment, and control group only receiving guideline-based treatment.

Study outcome: The primary outcome is favorable functional outcome, defined as modified Rankin Scale 0-2 at 90 days.

Keywords

Head-down position, acute ischemic stroke, protocol

Introduction

Currently, the guideline recommended reperfusion treatments such as intravenous thrombolysis and mechanical thrombectomy as the most effective treatment for acute ischemic stroke[1]. However, these treatments are limited by a strict time window and technique requirements, which obviously decreased eligible patients for reperfusion treatments.

Good collateral circulation has been demonstrated to provide compensatory blood supply to rescue the ischemic penumbra and reduce the infarct volume[2], which in turn improves the prognosis. How to effectively and safely improve collateral circulation remains a significant clinical challenge. The effect of head position on stroke has been investigated[3]. It is generally accepted that lying-flat position may increase blood flow and improved oxygenation[4]. The Head Positioning in Acute Stroke Trial (HeadPoST) is the first big sample study to compare the effects of the lying-flat versus sitting-up position in AIS patients[5], and the results showed that the lying-flat position was safe, but ineffective. The negative results may be due to the broad inclusion of stroke patients, and we argue that the patients with large artery atherosclerosis (LAA) etiology should be benefited from head position intervention.

In theory, compared with lying-flat position, the aggressive head-down position (i.e., fully supine Trendelenburg[6]) would more significantly increase blood flow to the ischemic penumbra and improve oxygenation of brain in the first hours or days after stroke[7]. Our recent results of animal and preliminary clinical studies showed that head-down position (HDP) may significantly improve neurological function.

Given that HDP is simple and clinically easy to operate, and may increase brain perfusion and improve collateral circulation in theory, we designed the prospective, multi-center, open-label, blinded-endpoint, randomized control study, aiming to explore the efficacy and safety of HDP for acute moderate anterior circulation stroke patients with large artery atherosclerosis within 24 hours of onset.

Methods

Design

HOPES2 is a prospective, multi-center, open-label, blind-endpoint, randomized control study to assess the efficacy and safety of HDP in moderate anterior circulation stroke patients with large artery atherosclerosis (LAA). The main purpose of this study is to test the hypothesis that two weeks' HDP therapy within 24 hours from symptom onset exerts neuroprotective effect on AIS patients with moderate deficit and LAA etiology.

Intervention

In this trial, eligible patients will be randomly assigned (1:1) using a computer-generated randomization sequence with block size of four and sealed envelopes, prepared by an independent statistician, into either HDP group receiving Trendelenburg (-20 degree) as an adjunct to guideline-based treatment, or control group only receiving guideline-based treatment (Figure 1). In the HDP group, the subjects are lowered to -20 degree in a supine position between 8:00-22:00 within 24h post-randomization as long as possible. During the procedure, when subjects feel intolerable, the head position will be elevated to lying flat (0 degree) for 5-10 minutes, and then the above procedure is repeated. After 24h post-randomization, the head-down procedures with -20 degree in a supine position with duration of 1-1.5 hour will be performed three times daily at 9:00-11:00, 15:00-17:00 and 20:00-22:00, respectively. In the control group, patients will be treated according to AHA/ASA guidelines for early management of ischemic stroke without any intervention of head position[1].

Figure 2. Study schema

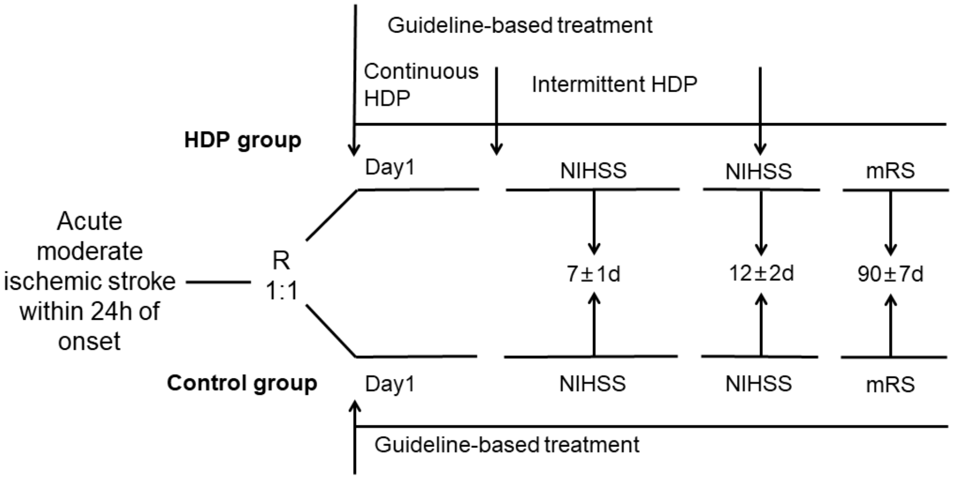

HDP: head-down position. mRS: modified Rankin Scale. NIHSS: National Institutes of Health Stroke Scale.

Patient population

A total of 100 patients with acute moderate ischemic stroke in ten centers are expected in China between December 2018 and December 2021. There are 50 subjects in the experimental group and control group, respectively. The detailed inclusion/exclusion criteria are listed in Table 1.

Table 2. The inclusion/exclusion criteria

**Inclusion criteria:**
1) Patient age ≥ 18 years.
2) Acute ischemic stroke confirmed by CT or MRI
3) The time from onset to treatment: within 24 h
4) Moderate neurological deficit: 6 ≤ National Institute of Health stroke scale (NIHSS) ≤ 16.
5) Large artery atherosclerosis etiology based on the Trial of Org 10172 in Acute Stroke Treatment (TOAST) criteria (culprit artery stenosis ≥ 50%, confirmed by CTA or MRA).
6) Evidence of unilateral large-vessel stenosis or occlusion of the internal carotid artery or middle cerebral artery (MCA) M1 or proximal M2 segment.

7)	first stroke onset or past stroke without obvious neurological deficit (mRS≤1)
8)	Signed informed consent from the patients, or their legally authorized representative.
**Exclusion criteria:**	
1)	Patients with disturbance of consciousness.
2)	Patients who plan to undergo or have completed thrombolysis or mechanical thrombectomy.
3)	Hemorrhagic stroke or combined stroke.
4)	Complicated with serious diseases, such as liver and kidney insufficiency, malignant tumor, etc.
5)	A history of stroke with severe sequelae (mRS ≥ 2).
6)	Other etiologies, such as cardiogenic embolism, arteritis, arterial dissection, moyamoya disease, etc.
7)	Previous history of intracerebral hemorrhage within 1 year.
8)	Any contraindication to head-down position (e.g. active vomiting, pneumonia, uncontrolled heart failure).
9)	Planned carotid or intracranial revascularization within 3 months.
10)	Enrolled in other clinical trials within 3 months.
11)	Pregnant or lactating women.
12)	Any inappropriate patient assessed by the researcher.

mRS: modified Rankin Scale; NIHSS: National Institutes of Health Stroke Scale.

Standard protocol approvals, registrations, and patient consents

The trial was registered on clinicaltrial.gov (NCT03744533). The protocol and data collection of the trial have been approved by the ethics committee of General Hospital of Shenyang Military Region and all participating sites. All subjects or their representatives will provide written informed consents before inclusion into the trial.

Data collection and follow-up

Demography and clinically relevant data are recorded at the time of entry into the study. All enrolled patients have face-to-face or telephone interview at baseline, 7 days, 12 days, and 90 days post-randomization, respectively. Data are recorded, identified, and assessed by experienced neurologists blinded to the intervention. Baseline and follow-up NIHSS scores were evaluated by the same neurologist who were not blinded to treatment allocation. Final 90-day mRS was evaluated by one qualified personnel who were blinded to treatment allocation according to a standardized procedure manual in each study center. To ensure validity and reproducibility of the evaluation, we held a training course for all investigators at each center. Concomitant medications and adverse events within 90 days after randomization will be recorded in detail by investigators.

Outcomes

The primary endpoint is the proportion of modified Rankin Scale (mRS) 0-2 at 90 days. The secondary endpoints are as follows: (1) proportion of mRS 0-1 at 90 days; (2) distribution of mRS score at 90 days; (3) proportion of early neurological deterioration (END), defined as more than 4 increase in National Institute of Health stroke scale (NIHSS) within 48 hours, but not result of cerebral hemorrhage; (4) change in NIHSS score compared with baseline at 12 days; (5) occurrence of stroke or other vascular events at 90 days; (6) proportion of death due to any cause within 90 days.

Quality control

Before the beginning of the study, all the investigators at each center attended training sessions to review the protocol and procedures. An independent Data Monitoring Committee (DMC) will perform to assure fidelity of conduct of the study according to the protocol and Good Clinical Practice (GCP). The primary purpose of the termination is to protect the rights and interests of the subjects and to avoid unnecessary economic losses. If the therapy shows a statistically significant difference of efficacy and/or safety over the other, the DMC has the right to terminate the study unconditionally.

Data management and monitoring

Data will be stored in the case report form (CRF). DMC is established to ensure ongoing monitoring of data security, such as hemorrhagic events and other adverse events, etc. Clinical outcome events (stroke, death, intracerebral hemorrhage, cardiopulmonary events, and stroke-associated pneumonia) will also be adjudicated by the independent DMC. Neuroimaging associated with clinical events will be read locally, reports will be included in adjudication packets, and actual images will be sent to the central reader from local investigators.

All AEs monitoring

All information about AEs should be recorded on the AEs page of the case report. All relevant SAEs are reviewed and adjudicated centrally in order to ensure that they meet the same diagnostic criteria.

Sample size determination

No formal sample size calculation was performed due to no relevant data from previous trial. For this exploratory trial, the sample size (50 patients per group) was determined primarily based on the suggestion of the Steer Committee.

Statistical analysis

All efficacy analyses will be performed according to the intention-to-treat principle, which comprises patients who received the allocated treatment and completed the assessment period. Baseline characteristics and procedural details will be compared with Student's t test if normally distributed or Wilcoxon test if non-normally distributed. Treatment effect will be presented as odds ratio (95% CI) of HDP group versus control group, analyzed by binary logistic regression. Shift analysis of the mRS scores at 90 days will be performed using ordinal logistic regression. Change in NIHSS score at 12 days from baseline will be calculated with generalized estimating equation. A sensitivity analysis will be undertaken for the key outcomes adjusted for confounding

covariates (age; NIHSS score at randomization; and the degree of vascular stenosis). Descriptive statistics of proportions will be used for the safety data. Continuous data are presented as mean (SD) or median (IQR) as appropriate. For categorical variables, absolute and relative frequencies are presented. there is statistical significance if *P* value < 0.05. Analyses will be performed with statistical software of IBM SPSS Statistics 24.

Study organization and funding

The protocol was designed by Hui-Sheng Chen and discussed by the academic team. Steering Committee is made up of external scientific advisors, and will monitor the research and data regularly. The study is supported by grants from the National Natural Science Foundation of the Peoples Republic of China (8207147) and the Science and Technology Project Plan of Liao Ning Province (2018225023, 2019JH2/10300027).

Discussion

It is recommended by guidelines that reperfusion therapies such as intravenous thrombolysis and mechanical thrombectomy can provide compensatory blood supply to save the ischemic penumbra and reduce the infarct volume, which improves the prognosis for acute ischemic stroke.[1] Up to date, there is a lack of effective method to improve cerebral perfusion and collateral circulation except reperfusion treatments.

The effect of head position on stroke has been investigated. For example, head position has an impact on cerebral blood flow, cerebral perfusion, and intracranial pressure.[8-10] The HeadPoST found that lying-flat position did not improve neurological function, compared with the sitting-up position in AIS patients.[5]

The current study is the first clinical trial to investigate the effect of HDP (-20 degree) on functional outcome in AIS patients, which is quite different from these previous studies. Firstly, head-down position with-20 degree was adopted in this study. In theory, compared with lying-flat position, the aggressive HDP would more significantly increase blood flow in the ischemic penumbra and improve oxygenation of brain in the initial hours or days after stroke onset.[11] The intervention strategy was further supported by our recent unpublished data that HDP with -20 degrees and one hour duration after

ischemia-reperfusion can improve neurological function and reduce infarct volume in rats with middle cerebral artery occlusion model, and reversed early neurological deterioration and improved clinical outcomes in several cases with LAA. Secondly, the current study will enroll acute moderate ischemic stroke patients with LAA, while the HeadPoST study did not classify the severity of cerebral infarction and etiology. We argue that these patients should be most likely to benefit from neuroprotective therapy, because the neuroprotective effect will be underestimated in patients with mild neurological deficit, while the patients with severe neurological deficit who was mostly due to large artery occlusion would not be improved by neuroprotective treatment without the help of reperfusion treatment. In addition, the pathogenesis of LAA is mostly related to hypoperfusion, whose neurological function would be improved from increased cerebral perfusion due to head-down position. Thirdly, long head position intervention strategy will be used in the current study: constant HDP for 10 hours within 24 hours followed by HDP three times daily for 2 weeks, while only first 24 hours after randomization in HeadPoST study. We argue that long-term head position intervention may result in more benefit of neuroprotective effect.

In conclusion, we conduct a prospective, open label, blinded endpoint, multi-center, randomized control trial to explore the efficacy and safety of HDP with about two weeks' duration in acute moderate ischemic stroke patients with large artery atherosclerosis within 24 hours from onset.

References

1 Powers, W. J., Rabinstein, A. A., Ackerson, T., Aet al. 2018 Guidelines for the Early Management of Patients With Acute Ischemic Stroke: A Guideline for Healthcare Professionals From the American Heart Association/American Stroke Association. *Stroke* **49:** e46–e110.

2 Shuaib A, Butcher K, Mohammad AA, Saqqur M, Liebeskind DS. Collateral blood vessels in acute ischaemic stroke: a potential therapeutic target. *Lancet Neurol*.2011; **10:** 909-21.

3 Olavarría VV, Arima H, Anderson CS, et al. Head position and cerebral blood flow velocity in acute ischemic stroke: a systematic review and meta-analysis. *Cerebrovasc Dis* 2014; **37**: 401-8.

4 Olavarría VV, Lavados PM, Muñoz-Venturelli P, et al. Flat-head positioning increases cerebral blood flow

in anterior circulation acute ischemic stroke. A cluster randomized phase IIb trial. *Int J Stroke* 2018; **13**: 600-11.

5 Anderson CS, Arima H, Lavados P, et al. Cluster-Randomized, Crossover Trial of Head Positioning in Acute Stroke. *N Engl J Med* 2017; **376**: 2437-47.

6 Martin JT. The Trendelenburg position: a review of current slants about head down tilt. *AANA J* 1995; **63**: 29-36.

7 Ogawa Y, Yanagida R, Ueda K, Aoki K, Iwasaki K. The relationship between widespread changes in gravity and cerebral blood flow. *Environ Health Prev Med* 2016; **21**: 186-92.

8 Favilla CG, Mesquita RC, Mullen M, et al. Optical bedside monitoring of cerebral blood flow in acute ischemic stroke patients during head-of-bed manipulation. *Stroke* 2014; **45**: 1269-74.

9 Schwarz S, Georgiadis D, Aschoff A, Schwab S. Effects of body position on intracranial pressure and cerebral perfusion in patients with large hemispheric stroke. *Stroke* 2002; **33**: 497-501.

10 Lu SS, Ge S, Su CQ, et al. MRI of plaque characteristics and relationship with downstream perfusion and cerebral infarction in patients with symptomatic middle cerebral artery stenosis. *J Magn Reson Imaging* 2018; **48**: 66-73.

11 Ax M, Sanchez-Crespo A, SGE L, Mure M, Petersson J. The influence of gravity on regional lung blood flow in humans: SPECT in the upright and head-down posture. *J Appl Physiol (1985)* 2017; **122**: 1445-51.

12 Lee SJ, Lee DG. Distribution of atherosclerotic stenosis determining early neurologic deterioration in acute ischemic stroke. *PLoS One* 2017; **12:** e0185314.

13 Kim SJ, Seok JM, Bang OY, et al. MR mismatch profiles in patients with intracranial atherosclerotic stroke: a comprehensive approach comparing stroke subtypes. *J Cereb Blood Flow Metab* 2009; **29:** 1138-45.

Supplementary Note 3: Summary of changes

The following is a list of main protocol changes from protocol version 1.0 dated 20 November 2018 to version 2.0 dated 19 March 2019.

The main reasons for the protocol changes in this series of amendments are:

Sections	Protocol version 1.0 change from	Protocol version 2.0 change to	Rationale
Abstract (study outcome)	The primary outcome is good functional outcome, defined as modified Rankin Scale 0-1 at 90 days.	The primary outcome is good functional outcome, defined as modified Rankin Scale 0-2 at 90 days.	Given that the neurological deficit of included subjects was relatively serious, we suggested that excellent functional outcome (modified Rankin Scale scoring 0-1 at 90 days) was more appropriate as secondary objectives than favorable functional outcome (modified Rankin Scale scoring 0-2 at 90 days).
Methods (inclusion criteria)	Moderate neurological deficit: 6 ≤ National Institute of Health stroke scale (NIHSS) ≤ 22	Moderate neurological deficit: 6 ≤ National Institute of Health stroke scale (NIHSS) ≤ 16	Given that subjects with serve neurological deficit (NIHSS > 16) maybe not benefit from HDP treatment, we suggested that subjects with NIHSS 6-16 were more appropriate to the HDP treatment.
Methods (outcomes)	(1) The primary endpoint is the proportion of modified Rankin Scale (mRS) 0-1 at 90 days.<br>(2) The secondary endpoints are as follows: (1) proportion of mRS 0-2 at 90 days;	(1) The primary endpoint is the proportion of modified Rankin Scale (mRS) 0-2 at 90 days.<br>(2) The secondary endpoints are as follows: (1) proportion of mRS 0-1 at 90 days;	Given that the neurological deficit of included subjects was relatively serious, we suggested excellent functional outcome (modified Rankin Scale scoring 0-1 at 90 days) was more appropriate as secondary objectives than favorable functional outcome (modified Rankin Scale scoring 0-2 at 90 days).

Supplementary Note 3: CONSORT checklist

Section/Topic	Item No	Checklist item	Reported on page No
**Title and abstract**			
	1a	Identification as a randomised trial in the title	1
	1b	Structured summary of trial design, methods, results, and conclusions (for specific guidance see CONSORT for abstracts)	3
**Introduction**			
Background and objectives	2a	Scientific background and explanation of rationale	5
	2b	Specific objectives or hypotheses	5
**Methods**			
Trial design	3a	Description of trial design (such as parallel, factorial) including allocation ratio	6
	3b	Important changes to methods after trial commencement (such as eligibility criteria), with reasons	NA
Participants	4a	Eligibility criteria for participants	6
	4b	Settings and locations where the data were collected	6
Interventions	5	The interventions for each group with sufficient details to allow replication, including how and when they were actually administered	7
Outcomes	6a	Completely defined pre-specified primary and secondary outcome measures, including how and when they were assessed	8
	6b	Any changes to trial outcomes after the trial commenced, with reasons	NA
Sample size	7a	How sample size was determined	8
	7b	When applicable, explanation of any interim analyses and stopping guidelines	NA
Randomisation:			
Sequence generation	8a	Method used to generate the random allocation sequence	7

	8b	Type of randomisation; details of any restriction (such as blocking and block size)	NA
Allocation concealment mechanism	9	Mechanism used to implement the random allocation sequence (such as sequentially numbered containers), describing any steps taken to conceal the sequence until interventions were assigned	7
Implementation	10	Who generated the random allocation sequence, who enrolled participants, and who assigned participants to interventions	7
Blinding	11a	If done, who was blinded after assignment to interventions (for example, participants, care providers, those assessing outcomes) and how	7
	11b	If relevant, description of the similarity of interventions	NA
Statistical methods	12a	Statistical methods used to compare groups for primary and secondary outcomes	8-9
	12b	Methods for additional analyses, such as subgroup analyses and adjusted analyses	9
**Results**			
Participant flow (a diagram is strongly recommended)	13a	For each group, the numbers of participants who were randomly assigned, received intended treatment, and were analysed for the primary outcome	20
	13b	For each group, losses and exclusions after randomisation, together with reasons	9
Recruitment	14a	Dates defining the periods of recruitment and follow-up	9
	14b	Why the trial ended or was stopped	NA
Baseline data	15	A table showing baseline demographic and clinical characteristics for each group	21
Numbers analysed	16	For each group, number of participants (denominator) included in each analysis and whether the analysis was by original assigned groups	9
Outcomes and estimation	17a	For each primary and secondary outcome, results for each group, and the estimated effect size and its precision (such as 95% confidence interval)	10
	17b	For binary outcomes, presentation of both absolute and relative effect sizes is recommended	10
Ancillary analyses	18	Results of any other analyses performed, including subgroup analyses and adjusted analyses, distinguishing pre-specified from exploratory	10
Harms	19	All important harms or unintended effects in each group (for specific guidance see CONSORT for harms)	11

**Discussion**			
Limitations	20	Trial limitations, addressing sources of potential bias, imprecision, and, if relevant, multiplicity of analyses	13-14
Generalisability	21	Generalisability (external validity, applicability) of the trial findings	11
Interpretation	22	Interpretation consistent with results, balancing benefits and harms, and considering other relevant evidence	11-13
**Other information**			
Registration	23	Registration number and name of trial registry	3
Protocol	24	Where the full trial protocol can be accessed, if available	Appendix 2
Funding	25	Sources of funding and other support (such as supply of drugs), role of funders	14

Supplementary Note 4: Original statistical analysis plan (v 1.0)

HEAD-DOWN POSITION FOR ACUTE MODERATE ISCHAEMIC STROKE WITH LARGE RTERY ATHEROSCLEROSIS: A PROSPECTIVE, RANDOM, OPEN LABEL, BLINDED END POINT, MULTI-CENTER STUDY

STATISTICAL ANALYSIS PLAN

ClinicalTrials.gov registration number: NCT03744533

Protocol version and date: Version 1.0, 25th November 2018

Chief Investigators: Prof Hui-Sheng Chen

Trial statisticians: Ms Nannan Zhang, Prof Duolao Wang

SAP authors: Ms Nannan Zhang, Prof Duolao Wang, Prof Hui-Sheng Chen

**SAP version history**		
Version Date	SAP Version #	Details of Changes
25 November 2018	*1.0*	*First version*

**Signatures**		
	Signature	Date
Ms Nannan Zhang (Trial Statistician)		25/11/2018
Prof Duolao Wang (Trial Statistician)		
Prof Hui-Sheng Chen (Chief Investigator)		
Prof Yi-Long Wang (IDMC Chair)		

TABLE OF CONTENTS

1. ABBREVIATIONS 4

2. INTRODUCTION 5
   2.1. Purpose of the statistical analysis plan 5
   2.2. Background to the study 5

3. STUDY OBJECTIVES AND OUTCOMES 5
   3.1. Study Objectives 5
      3.1.1. Primary Objective 5
      3.1.2. Secondary Objectives 5
   3.2. Outcomes 6
      3.2.1. Primary outcome 6
      3.2.2. Secondary outcomes 6
      3.2.3. Case ascertainment and case definitions 6

4. STUDY DESIGN 7
   4.1. Design 7
   4.2. Trial Sites 7
   4.3. Treatments 8
   4.4. Randomisation 8
   4.5. Sample Size 8

5. ANALYSIS POPULATIONS 8
   5.1. Study population data sets 8
   5.2. Analysis Close Date 8
   5.3. Data cleaning 9

6. STATISTICAL ANALYSES 9
   6.1. Primary Outcome Analysis 9
      6.1.1. mITT analysis of the primary outcome - the primary analysis 9
      6.1.2. Covariate adjusted analysis of the primary outcome 9
   6.2. Secondary Outcome Analysis 10
      6.2.1. Analysis of binary outcomes 10
      6.2.2. Analysis of time-to-event outcomes 10
      6.2.3. Analysis of secondary outcomes with repeated measurements 10
   6.3. Exploratory Analysis 10

7. SAFETY ANALYSES 111
   7.1. Safety Variables 111

8. GENERAL CONSIDERATIONS FOR DATA ANALYSES 11
   8.1. Covariates Analyses 11
   8.2. Multiplicity 122
   8.3. Missing data 12
   8.4. Further Exploratory Analyses 12
   8.5. Data Summaries 12

9. REFERENCE 13

ABBREVIATIONS

**Abbreviation**	**Explanation**
AE	Adverse Event
AIS	Acute ischaemic stroke
CI	Confidence Interval
CRF	Case Report Form
HDP	Head down position
IDMC	Independent Data Monitoring Committee
mITT	Modified intent-to-treat
LAA	Large artery atherosclerosis
mRS	Modified Rankin Scale
NIHSS	National Institute of Health Stroke Scale
OR	Odds Ratio
PP	Per-protocol
SAE	Serious Adverse Event
SAP	Statistical Analysis Plan
TMG	Trial Management Group

INTRODUCTION

Purpose of the statistical analysis plan

The purpose of this Statistical Analysis Plan (SAP) is to define the outcome variables, statistical methods, and analysis strategies to address the study's objectives in a prospective, random, open label, blinded end point, multi-center study to explore the efficacy and safety of head down position (HDP) for acute moderate anterior circulation stroke patients with large artery atherosclerosis (LAA) within 24 hours of onset. (Protocol version 1.0, 20/11/2018).

Background to the study

To date, there is no effective neuroprotection for acute ischemic stroke (AIS), except reperfusion strategy such as intravenous thrombolysis and mechanical thrombectomy [1-2]. The effect of head position as a nonpharmacological therapy on stroke has been investigated [3-4]. Previous studies suggested that lying-flat position may increase cerebral blood flow and improved oxygenation [5-6]. But no neuroprotective effect has been found. The Head Positioning in Acute Stroke Trial (HeadPoST) is the first big sample study to compare the effects of the lying-flat versus sitting-up position in AIS patients, but the results showed that the lying-flat position was safe, but ineffective. As a recent comment on HeadPoST pointed, the negative results may be due to the broad inclusion of stroke patients, which was a key critique of the trial, for example mostly with milder deficits and all stroke etiology, and the suitable patients may be those with large artery atherosclerosis (LAA) etiology.

In theory, compared with lying-flat position, the aggressive head down position (HDP) would more significantly increase blood flow to the ischemic penumbra [7]. Our recent results of animal and preliminary clinical studies showed that HDP may significantly improve neurological function.

Based on the above discussion, the prospective, random, open label, blinded endpoint, multi-center study is designed to explore the efficacy and safety of HDP for acute moderate anterior circulation stroke patients with LAA within 24 hours of onset.

STUDY OBJECTIVES AND OUTCOMES

Study Objectives

Primary Objective

To test the hypothesis that two weeks' HDP therapy clearly exerts the neuroprotective effect on patients with acute moderate ischemic stroke at 90 days, and has the good safety and tolerance.

Secondary Objectives

1. To determine the proportion of favorable functional outcome at 90 days by treatment group.

2. To determine the distribution of mRS (modified Rankin scale) at 90 days by treatment group.
3. To determine occurrence of early neurological deterioration within 48 hours by treatment group.
4. To determine change in neurological function at 12 days by treatment group.
5. To determine occurrence of stroke or other vascular events at 90 days by treatment group.
6. To determine all-cause mortality at 90 days by treatment group.
7. To determine HDP-related safety outcomes at 12 days by treatment group.

Outcomes

Primary outcome

The primary outcome is the occurrence of mRS (0-1) at 90 days (binary outcome), defined as a score of 0–1 on the mRS for the evaluation of neurological disability assessed in person or, if an in-person visit was not possible, by personnel certified in the scoring of the mRS at 90 days after randomisation through telephone.

Secondary outcomes

1. Occurrence of mRS (0-2) at 90 days (binary outcome)
2. Scores of mRS at 90 days (ordinal outcome)
3. Occurrence of early neurological deterioration (binary outcome)
4. Occurrence of safety outcomes included any adverse events and serious adverse events during HDP, such as fear, headache, anxiety, and intracranial hemorrhage (binary outcome)
5. Change in NIHSS score compared with baseline at 12 days (continuous outcome)
6. Time from randomisation to the occurrence of stroke or other vascular events at 90 days (time-to-event outcome)
7. Time from randomisation to the occurrence of death of any cause at 90 days (time-to-event outcome)

Case ascertainment and case definitions

(1) Deaths

All deaths during the study period will be recorded. Cause of death will be clinically ascertained by the study physicians (participants will not receive post-mortems). Mortality by treatment group will be analysed with all-cause mortality within 90 days as the secondary outcome.

(2) Early neurological deterioration

Early neurological deterioration was defined as more than 4 NIHSS scores increase within 48 hours, but not result of cerebral hemorrhage [8].

(3) intracranial hemorrhage

Intracranial hemorrhage was defined according to the ECASS-1 study [9].

(4) Stroke

Stroke was defined as an acute focal central neurological deficit lasting >24 hours that resulted in irreversible brain damage or body impairment by a vascular cause [10].

(5) Other vascular events

Other vascular events include pulmonary embolism, peripheral vessel incident, and cardiovascular incident, which was not present at the beginning of the study.

(6) Additional Safety Variables

Adverse events (AE) is any adverse medical event that occurs in the course of the study. All information about AEs should be recorded including fear, headache, anxiety, which was not present at the beginning of the study, and whether the unexpected AE is associated with the HDP will be further adjudicated by principal investigator.

STUDY DESIGN

Design

This is a prospective, random, open label, blinded end point, multi-center trial in patients with acute moderate ischaemic stroke.

Trial Sites

Trial recruitment will take place at ten hospitals nationwide. The trial sites build on prior successful collaborations, and have been selected due to their proven ability to successfully execute clinical trials of acute ischaemic stroke, and to reflect a spectrum of China health care settings.

- Department of Neurology, Beipiao Central Hospital, Beipiao, China
- Department of Neurology, Panjin Central Hospital, Panjin, China
- Department of Neurology, Chaoyang Central Hospital, Chaoyang, China
- Department of Neurology, General Hospital of Northern Theater Command, Shenyang, China
- Department of Neurology, Dandong First Hospital, Dandong, China
- Department of Neurology, The Affiliated Central Hospital of Shenyang Medical College, Shenyang, China
- Department of Neurology, Lvshun Chinese Medicine Hospital, Dalian, China
- Department of Neurology, Anshan Central Hospital, Anshan, China
- Department of Neurology, Anshan Changda Hospital, Anshan, China

- Department of Neurology, Fukuang General Hospital of Liaoning Health Industry Group, Fushun, China

Treatments

Trial arms:

The study regimens are:

HDP group: guideline-based therapy and HDP, which was given continuously HDP within 24 hours and three times a day with -20 degree Trendelenburg for 10 to 14 days.

Control group: guideline-based therapy for 10 to 14 days.

Randomisation

In this trial, participants will be randomised in a 1:1 ratio using a computer-generated randomisation sequence with block size of four and sealed envelopes.

Sample Size

No formal sample size calculation was performed due to no relevant data from previous trial. For this exploratory trial, the sample size (50 patients per group) was determined primarily based on the suggestion of the Steer Committee.

ANALYSIS POPULATIONS

Study population data sets

The membership of each analysis set will be determined and documented and the reasons for exclusion will be given prior to database lock. A summary table will list the individual subjects sorted by treatment group and describe their protocol deviation/violation.

Modified Intent-to-Treat (mITT) population

All participants with valid informed consent will be included in the mITT population according to the treatment to which they are randomised, regardless of whether they prematurely discontinue treatment or are otherwise protocol violators/deviators. Participants lost to follow-up or withdrawn will not be included in the mITT population.

Analysis Close Date

The analysis close date is the date on which the last participant completed 90-day follow-up.

Last contact date (also referred to as Trial reference end date): the date of the last trial related procedure. For survival subjects it is defined as the maximum of

- Date of last office visit (scheduled or unscheduled visit)

- Date of the last follow-up contact (including last date on subject survival status recorded)

- Date of the last known adverse event (AE) status or lab results reported on the AE or lab case report from (CRF) pages, respectively

Data cleaning

The data will then be checked to ensure that there are no erroneous entries and that all missing data is properly coded. Any changes will be made on the paper CRF.

STATISTICAL ANALYSES

The analyses will be carried out by the trial statistician and the primary analysis will be reviewed by a second statistician. The principle of mITT will be the main strategy of the analysis adopted for the primary outcome and all the secondary outcomes.

Primary Outcome Analysis

mITT analysis of the primary outcome - the primary analysis

The primary outcome is a binary outcome: excellent functional outcome defined as mRS (0-1) at 90 days. The primary analysis will be based on the mITT population as defined above.

The primary endpoint will be summarised by number (%) of participants that have excellent functional outcome by treatment group. A formal statistical analysis will be performed as a binary logistic regression. In the binary logistic regression model, the occurrence of favorable functional outcome at 90 days will be treated as the response variable and the treatment as the only predictor. From this model, odds ratio of having a primary outcome between HDP and Control together with two-sided 95% confidence interval (CI) and p-value will be derived.

Covariate adjusted analysis of the primary outcome

Adjusted analyses will also be carried out on the analysis of the primary endpoint to determine whether the treatment effect estimate is affected with the inclusion of covariables. The covariables that will be included in the adjusted analyses are:

- Age
- NIHSS score at randomisation
- The degree of vascular stenosis

From the above model, the adjusted odds ratio (OR) and 95% CI comparing the HDP to the Control will be derived.

The above binary logistic regression model may not converge when all covariates are introduced into the model simultaneously. To avoid non-convergence issue, we will first calculate a propensity score with treatment as the dependent variable (1 for HDP and 0 for Control) and all covariates listed above as independent variables through a logistic

regression model, and then include the calculated propensity score (continuous variable) as a covariate in the logistic regression model.

Imputation for baseline missing covariates (see description below **8.3 missing data**) will be made for covariate adjusted analysis.

Secondary Outcome Analysis

Secondary outcome analyses will be based on the mITT populations.

Analysis of binary outcomes

Proportion of mRS (0-2) at 90 days and occurrence of early neurological deterioration within 48 hours will be treated as a binary outcome and will be summarised by number (%) of participants with event by treatment group and analysed in a similar way as the primary endpoint by means of binary logistic regression. The OR and its two-sided 95% CI between HDP and Control will be estimated.

The analysis of other binary outcomes will also use binary logistic regression models with treatment as the only predictor. ORs with their two-sided 95% CIs comparing two treatment arms will be derived from the binary logistic regression models.

Analysis of time-to-event outcomes

The time-to-event outcomes (e.g. time from randomisation to the occurrence of death from any cause at the end of 90 days) will be summarised by number (%) of participants with event and incidence rate by treatment arm.

Survival curves will be plotted using Kaplan-Meier method and compared using the log-rank test. Cox regression model will be used to derive hazard ratio and its 2-sided 95%CI for comparing two treatment groups.

Analysis of secondary outcomes with repeated measurements

The NIHSS score is measured at admission and 12 days later.

These data will be managed according to the following procedures and rules before being analysed:

We will calculate the change of NIHSS score for each patient between randomisation and 12 days, and used a linear regression model to compare the means in the change from baseline between the 2 groups.

Exploratory Analysis

Other statistical methods may be used if deemed necessary but was considered as exploratrory.

SAFETY ANALYSES

Safety Variables

Adverse events (AEs) will be restricted to those occurring during the 90 days after randomisation.

AEs will be summarised using the number of AEs, the number (%) of participants with AEs by treatment arms. The number of patients with any AE or SAEs will be analysed using logistic regression model from which odds ratios and its 95%CI will be calculated.

Safety analyses will summarise the number of any adverse medical events, serious adverse events (SAEs), and deaths occurring after randomisation.

Summaries of the total number of reported AEs/SAEs and number of participants reporting at least one AEs/SAE will be presented by treatment received and overall. In addition, summaries of the suspected relationship with trial treatment, suspected trial treatment or other cause, duration of recovered SAEs, seriousness criteria, event outcome, DAIDS grade and SAE, will be presented by treatment received and overall.

Line listings of all reported SAEs for each participant will also be presented by treatment received. They will include (where appropriate):

- Randomised treatment
- DAIDS grade
- Event description
- Seriousness criteria
- Suspected relationship to the trial medications
- Suspected products
- Other causality
- Expectedness
- Date of randomisation
- Date of onset
- Date event became serious (serious events only)
- Date of recovery
- Outcome
- Details of the treatment received

GENERAL CONSIDERATIONS FOR DATA ANALYSES

SPSS® (version 23) will be used to perform all data analyses.

Covariates Analyses

Covariate analyses will be performed on the primary outcome and secondary outcomes on the mITT. Other covariate analyses will be performed if deemed necessary.

Multiplicity

Analyses of secondary outcomes and additional analyses for the primary outcome are regarded as exploratory in nature, therefore, multiplicity adjustment will not apply to the primary and secondary outcome analyses.

Missing data

Missing baseline covariates will be imputed using simple imputation methods in the covariate adjusted analysis based on the covariate distributions. For a continuous variable, missing values will be imputed with mean calculated from the available sample. For a categorical variable, missing values will be imputed with the most frequent value calculated from the sample.

Further Exploratory Analyses

Further exploratory analyses may be carried out should they be deemed necessary; this will be at the discretion of the TMG. These will be added to the analysis plan as an amendment along with justification, where appropriate.

Data Summaries

Continuous variables will be summarised according to number of subjects with non-missing data (n), mean, standard deviation (SD), median, minimum, and maximum. The confidence interval will be added on summaries of continuous effectiveness variables.

Categorical variables will be summarised according to the absolute frequency and percentage of subjects (%) in each category level. The denominator for the percentages is the number of subjects in the treatment arm with data available, unless noted otherwise. Event rates per 100 person years will also be reported for time-to-event clinical outcomes and adverse events of special interest.

REFERENCE

1. Ferro JM, Bousser MG, Canhão P, et al. European Stroke Organization guideline for the diagnosis and treatment of cerebral venous thrombosis - Endorsed by the European Academy of Neurology. *Eur Stroke J* 2017; **2:** 195-221.
2. Wang G, Fang B, Yu X, Li Z. Interpretation of 2018 guidelines for the early management of patients with acute ischemic stroke. *Zhonghua Wei Zhong Bing Ji Jiu Yi Xue* 2018; **30:** 289-95.
3. Olavarría VV, Arima H, Anderson CS, et al. Head position and cerebral blood flow velocity in acute ischemic stroke: a systematic review and meta-analysis. *Cerebrovasc Dis* 2014; **37:** 401-08.
4. Anderson CS, Arima H, Lavados P, et al. Cluster-Randomized, Crossover Trial of Head Positioning in Acute Stroke. *N Engl J Med* 2017; **376:** 2437-47.
5. Olavarría VV, Lavados PM, Muñoz-Venturelli P, et al. Flat-head positioning increases cerebral blood flow in anterior circulation acute ischemic stroke. A cluster randomized phase IIb trial. *Int J Stroke* 2018; **13:** 600-11.
6. Ogawa Y, Yanagida R, Ueda K, Aoki K, Iwasaki K. The relationship between widespread changes in gravity and cerebral blood flow. *Environ Health Prev Med* 2016; **21:** 186-92.
7. Martin JT. The Trendelenburg position: a review of current slants about head down tilt. *AANA J* 1995; **63:** 29-36.
8. National Institute of Neurological Disorders and Stroke rt-PA Stroke Study Group. Tissue plasminogen activator for acute ischemic stroke. *N Engl J Med*. 1995; **333:** 1581-7.
9. Hacke W, Kaste M, Fieschi C, et al. Intravenous thrombolysis with recombinant tissue plasminogen activator for acute hemispheric stroke. The European Cooperative Acute Stroke Study (ECASS). *JAMA* 1995; **274:** 1017-25.
10. Campbell BCV, Khatri P. Stroke. *Lancet* 2020; **396:** 129-42.

Supplementary Note 5: Final statistical analysis plan (v 2.0)

**HEAD-DOWN POSITION FOR ACUTE MODERATE ISCHAEMIC STROKE WITH LARGE RTERY ATHEROSCLEROSIS: A PROSPECTIVE, RANDOM, OPEN LABEL, BLINDED END POINT, MULTI-CENTER STUDY**

STATISTICAL ANALYSIS PLAN

ClinicalTrials.gov registration number: NCT03744533

Protocol version and date: Version 2.0, 19th March 2019

Chief Investigators: Prof Hui-Sheng Chen<br><br>***Trial statisticians:*** Ms Nannan Zhang, Prof Duolao Wang<br><br>***SAP authors:*** Ms Nannan Zhang, Prof Duolao Wang, Prof Hui-Sheng Chen

SAP version history		
Version Date	SAP Version #	Details of Changes
19 March 2019	*2.0*	*Second version*

**Signatures**		
	Signature	Date
Ms Nannan Zhang (Trial Statistician)		19/3/2019
Prof Duolao Wang (Trial Statistician)		
Prof Hui-Sheng Chen (Chief Investigator)		
Prof Yi-Long Wang (IDMC Chair)		

TABLE OF CONTENTS

1. ABBREVIATIONS 4

2. INTRODUCTION 5
   2.1. Purpose of the statistical analysis plan 5
   2.2. Background to the study 5

3. STUDY OBJECTIVES AND OUTCOMES 5
   3.1. Study Objectives 5
      3.1.1. Primary Objective 5
      3.1.2. Secondary Objectives 5
   3.2. Outcomes 6
      3.2.1. Primary outcome 6
      3.2.2. Secondary outcomes 6
      3.2.3. Case ascertainment and case definitions 6

4. STUDY DESIGN 7
   4.1. Design 7
   4.2. Trial Sites 7
   4.3. Treatments 8
   4.4. Randomisation 8
   4.5. Sample Size 8

5. ANALYSIS POPULATIONS 8
   5.1. Study population data sets 8
   5.2. Analysis Close Date 8
   5.3. Data cleaning 9

6. STATISTICAL ANALYSES 9
   6.1. Primary Outcome Analysis 9
      6.1.1. mITT analysis of the primary outcome - the primary analysis 9
      6.1.2. Covariate adjusted analysis of the primary outcome 9
   6.2. Secondary Outcome Analysis 10
      6.2.1. Analysis of binary outcomes 10
      6.2.2. Analysis of time-to-event outcomes 10
      6.2.3. Analysis of secondary outcomes with repeated measurements 10
   6.3. Exploratory Analysis 10

7. SAFETY ANALYSES 111
   7.1. Safety Variables 11

8. GENERAL CONSIDERATIONS FOR DATA ANALYSES 11
   8.1. Covariates Analyses 11
   8.2. Multiplicity 12
   8.3. Missing data 12
   8.4. Further Exploratory Analyses 12
   8.5. Data Summaries 12

9. REFERENCE 13

1. ABBREVIATIONS

**Abbreviation**	**Explanation**
AE	Adverse Event
AIS	Acute ischaemic stroke
CI	Confidence Interval
CRF	Case Report Form
HDP	Head down position
IDMC	Independent Data Monitoring Committee
mITT	Modified intent-to-treat
LAA	Large artery atherosclerosis
mRS	Modified Rankin Scale
NIHSS	National Institute of Health Stroke Scale
OR	Odds Ratio
PP	Per-protocol
SAE	Serious Adverse Event
SAP	Statistical Analysis Plan
TMG	Trial Management Group

2. INTRODUCTION

2.1. Purpose of the statistical analysis plan

The purpose of this Statistical Analysis Plan (SAP) is to define the outcome variables, statistical methods, and analysis strategies to address the study's objectives in a prospective, random, open label, blinded end point, multi-center study to explore the efficacy and safety of head down position (HDP) for acute moderate anterior circulation stroke patients with large artery atherosclerosis (LAA) within 24 hours of onset. (Protocol version 2.0, 19/3/2019).

2.2. Background to the study

To date, there is no effective neuroprotection for acute ischemic stroke (AIS), except reperfusion strategy such as intravenous thrombolysis and mechanical thrombectomy [1-2]. The effect of head position as a nonpharmacological therapy on stroke has been investigated [3-4]. Previous studies suggested that lying-flat position may increase cerebral blood flow and improved oxygenation [5-6]. But no neuroprotective effect has been found. The Head Positioning in Acute Stroke Trial (HeadPoST) is the first big sample study to compare the effects of the lying-flat versus sitting-up position in AIS patients, but the results showed that the lying-flat position was safe, but ineffective. As a recent comment on HeadPoST pointed, the negative results may be due to the broad inclusion of stroke patients, which was a key critique of the trial, for example mostly with milder deficits and all stroke etiology, and the suitable patients may be those with large artery atherosclerosis (LAA) etiology.

In theory, compared with lying-flat position, the aggressive head down position (HDP) would more significantly increase blood flow to the ischemic penumbra [7]. Our recent results of animal and preliminary clinical studies showed that HDP may significantly improve neurological function.

Based on the above discussion, the prospective, random, open label, blinded endpoint, multi-center study is designed to explore the efficacy and safety of HDP for acute moderate anterior circulation stroke patients with LAA within 24 hours of onset.

3. STUDY OBJECTIVES AND OUTCOMES

3.1. Study Objectives

3.1.1. Primary Objective

To test the hypothesis that two weeks' HDP therapy clearly exerts the neuroprotective effect on patients with acute moderate ischemic stroke at 90 days, and has the good safety and tolerance.

3.1.2. Secondary Objectives

1. To determine the proportion of excellent functional outcome at 90 days by treatment group.
2. To determine the distribution of mRS (modified Rankin scale) at 90 days by treatment group.

3. To determine occurrence of early neurological deterioration within 48 hours by treatment group.
4. To determine change in neurological function at 12 days by treatment group.
5. To determine occurrence of stroke or other vascular events at 90 days by treatment group.
6. To determine all-cause mortality at 90 days by treatment group.
7. To determine HDP-related safety outcomes at 12 days by treatment group.

3.2. Outcomes

3.2.1. Primary outcome

The primary outcome is the occurrence of mRS (0-2) at 90 days (binary outcome), defined as a score of 0–2 on the mRS for the evaluation of neurological disability assessed in person or, if an in-person visit was not possible, by personnel certified in the scoring of the mRS at 90 days after randomisation through telephone.

3.2.2. Secondary outcomes

1. Occurrence of mRS (0-1) at 90 days (binary outcome)
2. Scores of mRS at 90 days (ordinal outcome)
3. Occurrence of early neurological deterioration (binary outcome)
4. Occurrence of safety outcomes included any adverse events and serious adverse events during HDP, such as fear, headache, anxiety, and intracranial hemorrhage (binary outcome)
5. Change in NIHSS score compared with baseline at 12 days (continuous outcome)
6. Time from randomisation to the occurrence of stroke or other vascular events at 90 days (time-to-event outcome)
7. Time from randomisation to the occurrence of death of any cause at 90 days (time-to-event outcome)

3.2.3. Case ascertainment and case definitions

(1) Deaths

All deaths during the study period will be recorded. Cause of death will be clinically ascertained by the study physicians (participants will not receive post-mortems). Mortality by treatment group will be analysed with all-cause mortality within 90 days as the secondary outcome.

(2) Early neurological deterioration

Early neurological deterioration was defined as more than 4 NIHSS scores increase within 48 hours, but not result of cerebral hemorrhage [8].

(3) intracranial hemorrhage

Intracranial hemorrhage was defined according to the ECASS-1 study [9].

(4) Stroke

Stroke was defined as an acute focal central neurological deficit lasting >24 hours that resulted in irreversible brain damage or body impairment by a vascular cause [10].

(5) Other vascular events

Other vascular events include pulmonary embolism, peripheral vessel incident, and cardiovascular incident, which was not present at the beginning of the study.

(6) Additional Safety Variables

Adverse events (AE) is any adverse medical event that occurs in the course of the study. All information about AEs should be recorded including fear, headache, anxiety, which was not present at the beginning of the study, and whether the unexpected AE is associated with the HDP will be further adjudicated by principal investigator.

4. STUDY DESIGN

4.1. Design

This is a prospective, random, open label, blinded end point, multi-center trial in patients with acute moderate ischaemic stroke.

4.2. Trial Sites

Trial recruitment will take place at ten hospitals nationwide. The trial sites build on prior successful collaborations, and have been selected due to their proven ability to successfully execute clinical trials of acute ischaemic stroke, and to reflect a spectrum of China health care settings.

- Department of Neurology, Beipiao Central Hospital, Beipiao, China
- Department of Neurology, Panjin Central Hospital, Panjin, China
- Department of Neurology, Chaoyang Central Hospital, Chaoyang, China
- Department of Neurology, General Hospital of Northern Theater Command, Shenyang, China
- Department of Neurology, Dandong First Hospital, Dandong, China
- Department of Neurology, The Affiliated Central Hospital of Shenyang Medical College, Shenyang, China
- Department of Neurology, Lvshun Chinese Medicine Hospital, Dalian, China
- Department of Neurology, Anshan Central Hospital, Anshan, China
- Department of Neurology, Anshan Changda Hospital, Anshan, China

- Department of Neurology, Fukuang General Hospital of Liaoning Health Industry Group, Fushun, China

4.3. Treatments

Trial arms:

The study regimens are:

HDP group: guideline-based therapy and HDP, which was given continuously HDP within 24 hours and three times a day with -20 degree Trendelenburg for 10 to 14 days.

Control group: guideline-based therapy for 10 to 14 days.

4.4. Randomisation

In this trial, participants will be randomised in a 1:1 ratio using a computer-generated randomisation sequence with block size of four and sealed envelopes.

4.5. Sample Size

No formal sample size calculation was performed due to no relevant data from previous trial. For this exploratory trial, the sample size (50 patients per group) was determined primarily based on the suggestion of the Steer Committee.

5. ANALYSIS POPULATIONS

5.1. Study population data sets

The membership of each analysis set will be determined and documented and the reasons for exclusion will be given prior to database lock. A summary table will list the individual subjects sorted by treatment group and describe their protocol deviation/violation.

Modified Intent-to-Treat (mITT) population

All participants with valid informed consent will be included in the mITT population according to the treatment to which they are randomised, regardless of whether they prematurely discontinue treatment or are otherwise protocol violators/deviators. Participants lost to follow-up or withdrawn will not be included in the mITT population.

5.2. Analysis Close Date

The analysis close date is the date on which the last participant completed 90-day follow-up.

Last contact date (also referred to as Trial reference end date): the date of the last trial related procedure. For survival subjects it is defined as the maximum of

- Date of last office visit (scheduled or unscheduled visit)

- Date of the last follow-up contact (including last date on subject survival status recorded)
- Date of the last known adverse event (AE) status or lab results reported on the AE or lab case report from (CRF) pages, respectively

5.3. Data cleaning

The data will then be checked to ensure that there are no erroneous entries and that all missing data is properly coded. Any changes will be made on the paper CRF.

6. STATISTICAL ANALYSES

The analyses will be carried out by the trial statistician and the primary analysis will be reviewed by a second statistician. The principle of mITT will be the main strategy of the analysis adopted for the primary outcome and all the secondary outcomes.

6.1. Primary Outcome Analysis

6.1.1. mITT analysis of the primary outcome - the primary analysis

The primary outcome is a binary outcome: favorable functional outcome defined as mRS (0-2) at 90 days. The primary analysis will be based on the mITT population as defined above.

The primary endpoint will be summarised by number (%) of participants that have excellent functional outcome by treatment group. A formal statistical analysis will be performed as a binary logistic regression. In the binary logistic regression model, the occurrence of favorable functional outcome at 90 days will be treated as the response variable and the treatment as the only predictor. From this model, odds ratio of having a primary outcome between HDP and Control together with two-sided 95% confidence interval (CI) and p-value will be derived.

6.1.2. Covariate adjusted analysis of the primary outcome

Adjusted analyses will also be carried out on the analysis of the primary endpoint to determine whether the treatment effect estimate is affected with the inclusion of covariables. The covariables that will be included in the adjusted analyses are:

- Age
- NIHSS score at randomisation
- The degree of vascular stenosis

From the above model, the adjusted odds ratio (OR) and 95% CI comparing the HDP to the Control will be derived.

The above binary logistic regression model may not converge when all covariates are introduced into the model simultaneously. To avoid non-convergence issue, we will first calculate a propensity score with treatment as the dependent variable (1 for HDP and 0 for Control) and all covariates listed above as independent variables through a logistic regression model, and then include the calculated propensity score (continuous variable) as a covariate in the logistic regression model.

Imputation for baseline missing covariates (see description below **8.3 missing data**) will be made for covariate adjusted analysis.

6.2. Secondary Outcome Analysis

Secondary outcome analyses will be based on the mITT populations.

6.2.1. Analysis of binary outcomes

Proportion of mRS (0-1) at 90 days and occurrence of early neurological deterioration within 48 hours will be treated as a binary outcome and will be summarised by number (%) of participants with event by treatment group and analysed in a similar way as the primary endpoint by means of binary logistic regression. The OR and its two-sided 95% CI between HDP and Control will be estimated.

The analysis of other binary outcomes will also use binary logistic regression models with treatment as the only predictor. ORs with their two-sided 95% CIs comparing two treatment arms will be derived from the binary logistic regression models.

6.2.2. Analysis of time-to-event outcomes

The time-to-event outcomes (e.g. time from randomisation to the occurrence of death from any cause at the end of 90 days) will be summarised by number (%) of participants with event and incidence rate by treatment arm.

Survival curves will be plotted using Kaplan-Meier method and compared using the log-rank test. Cox regression model will be used to derive hazard ratio and its 2-sided 95%CI for comparing two treatment groups.

6.2.3. Analysis of secondary outcomes with repeated measurements

The NIHSS score is measured at admission and 12 days later.

These data will be managed according to the following procedures and rules before being analysed:

We will calculate the change of NIHSS score for each patient between randomisation and 12 days, and used a linear regression model to compare the means in the change from baseline between the 2 groups.

6.3. Exploratory Analysis

Other statistical methods may be used if deemed necessary but was considered as exploratrory.

7. SAFETY ANALYSES

7.1. Safety Variables

Adverse events (AEs) will be restricted to those occurring during the 90 days after randomisation.

AEs will be summarised using the number of AEs, the number (%) of participants with AEs by treatment arms. The number of patients with any AE or SAEs will be analysed using logistic regression model from which odds ratios and its 95%CI will be calculated.

Safety analyses will summarise the number of any adverse medical events, serious adverse events (SAEs), and deaths occurring after randomisation.

Summaries of the total number of reported AEs/SAEs and number of participants reporting at least one AEs/SAE will be presented by treatment received and overall. In addition, summaries of the suspected relationship with trial treatment, suspected trial treatment or other cause, duration of recovered SAEs, seriousness criteria, event outcome, DAIDS grade and SAE, will be presented by treatment received and overall.

Line listings of all reported SAEs for each participant will also be presented by treatment received. They will include (where appropriate):

- Randomised treatment
- DAIDS grade
- Event description
- Seriousness criteria
- Suspected relationship to the trial medications
- Suspected products
- Other causality
- Expectedness
- Date of randomisation
- Date of onset
- Date event became serious (serious events only)
- Date of recovery
- Outcome
- Details of the treatment received

8. GENERAL CONSIDERATIONS FOR DATA ANALYSES

SPSS® (version 23) will be used to perform all data analyses.

8.1. Covariates Analyses

Covariate analyses will be performed on the primary outcome and secondary outcomes on the mITT. Other covariate analyses will be performed if deemed necessary.

8.2. Multiplicity

Analyses of secondary outcomes and additional analyses for the primary outcome are regarded as exploratory in nature, therefore, multiplicity adjustment will not apply to the primary and secondary outcome analyses.

8.3. Missing data

Missing baseline covariates will be imputed using simple imputation methods in the covariate adjusted analysis based on the covariate distributions. For a continuous variable, missing values will be imputed with mean calculated from the available sample. For a categorical variable, missing values will be imputed with the most frequent value calculated from the sample.

8.4. Further Exploratory Analyses

Further exploratory analyses may be carried out should they be deemed necessary; this will be at the discretion of the TMG. These will be added to the analysis plan as an amendment along with justification, where appropriate.

8.5. Data Summaries

Continuous variables will be summarised according to number of subjects with non-missing data (n), mean, standard deviation (SD), median, minimum, and maximum. The confidence interval will be added on summaries of continuous effectiveness variables.

Categorical variables will be summarised according to the absolute frequency and percentage of subjects (%) in each category level. The denominator for the percentages is the number of subjects in the treatment arm with data available, unless noted otherwise. Event rates per 100 person years will also be reported for time-to-event clinical outcomes and adverse events of special interest.

9. REFERENCE

1) Ferro JM, Bousser MG, Canhão P, et al. European Stroke Organization guideline for the diagnosis and treatment of cerebral venous thrombosis - Endorsed by the European Academy of Neurology. *Eur Stroke J* 2017; **2:** 195-221.
2) Wang G, Fang B, Yu X, Li Z. Interpretation of 2018 guidelines for the early management of patients with acute ischemic stroke. *Zhonghua Wei Zhong Bing Ji Jiu Yi Xue* 2018; **30:** 289-95.
3) Olavarría VV, Arima H, Anderson CS, et al. Head position and cerebral blood flow velocity in acute ischemic stroke: a systematic review and meta-analysis. *Cerebrovasc Dis* 2014; **37:** 401-08.
4) Anderson CS, Arima H, Lavados P, et al. Cluster-Randomized, Crossover Trial of Head Positioning in Acute Stroke. *N Engl J Med* 2017; **376:** 2437-47.
5) Olavarría VV, Lavados PM, Muñoz-Venturelli P, et al. Flat-head positioning increases cerebral blood flow in anterior circulation acute ischemic stroke. A cluster randomized phase IIb trial. *Int J Stroke* 2018; **13:** 600-11.
6) Ogawa Y, Yanagida R, Ueda K, Aoki K, Iwasaki K. The relationship between widespread changes in gravity and cerebral blood flow. *Environ Health Prev Med* 2016; **21:** 186-92.
7) Martin JT. The Trendelenburg position: a review of current slants about head down tilt. *AANA J* 1995; **63:** 29-36.
8) National Institute of Neurological Disorders and Stroke rt-PA Stroke Study Group. Tissue plasminogen activator for acute ischemic stroke. *N Engl J Med*. 1995; **333:** 1581-7.
9) Hacke W, Kaste M, Fieschi C, et al. Intravenous thrombolysis with recombinant tissue plasminogen activator for acute hemispheric stroke. The European Cooperative Acute Stroke Study (ECASS). *JAMA* 1995; **274:** 1017-25.
10) Campbell BCV, Khatri P. Stroke. *Lancet* 2020; **396:** 129-42.

Supplementary Note 6: Summary of changes – Statistical analysis plan version 1.0 to version 2.0

The following is a list of main statistical analysis plan changes from protocol version 1.0 dated 20 November 2018 to version 2.0 dated 19 March 2019.

The main reasons for the statistical analysis plan changes in this series of amendments are:

Sections	Statistical analysis plan version 1.0 change from	Statistical analysis plan version 2.0 change to	Rationale
Introduction (purpose of the statistical analysis plan)	(Protocol version 1.0, 20/11/2018)	(Protocol version 2.0, 19/3/2019)	Change
Study objectives and outcomes (secondary objectives)	To determine the proportion of favourable functional outcome at 90 days by treatment group.	To determine the proportion of excellent functional outcome at 90 days by treatment group.	Given that the neurological deficit of included subjects was relatively serious, we suggested that excellent functional outcome (modified Rankin Scale scoring 0-1 at 90 days) was more appropriate as secondary objectives than favourable functional outcome (modified Rankin Scale scoring 0-2 at 90 days).
Outcomes (primary outcome)	The primary outcome is the occurrence of mRS (0-1) at 90 days (binary outcome), defined as a score of 0–1 on the mRS for the evaluation of neurological disability assessed in person or, if	The primary outcome is the occurrence of mRS (0-2) at 90 days (binary outcome), defined as a score of 0–2 on the mRS for the evaluation of neurological disability assessed in person or, if	Given that the neurological deficit of included subjects was relatively serious, we suggested that modified Rankin Scale scoring 0-2 at 90 days was more appropriate as primary

	an in-person visit was not possible, by personnel certified in the scoring of the mRS at 90 days after randomisation through telephone.	an in-person visit was not possible, by personnel certified in the scoring of the mRS at 90 days after randomisation through telephone.	outcome than modified Rankin Scale scoring 0-1 at 90 days.
Outcomes (secondary outcomes)	Occurrence of mRS (0-2) at 90 days (binary outcome)	Occurrence of mRS (0-1) at 90 days (binary outcome)	Given that the neurological deficit of included subjects was relatively serious, we suggested that modified Rankin Scale scoring 0-1 at 90 days was more appropriate as secondary outcome than modified Rankin Scale scoring 0-2 at 90 days.
Statistical analyses (primary outcome analysis-mITT analysis of the primary outcome - the primary analysis)	The primary outcome is a binary outcome: excellent functional outcome defined as mRS (0-1) at 90 days.	The primary outcome is a binary outcome: favourable functional outcome defined as mRS (0-2) at 90 days.	Given that the neurological deficit of included subjects was relatively serious, we suggested that modified Rankin Scale scoring 0-2 at 90 days was more appropriate as primary outcome than modified Rankin Scale scoring 0-1 at 90 days.
Statistical analyses (secondary outcome analysis- analysis of	Proportion of mRS (0-2) at 90 days and occurrence of early neurological deterioration within 48 hours will be treated as a binary outcome and will be summarised by number (%) of participants with event by treatment group and	Proportion of mRS (0-1) at 90 days and occurrence of early neurological deterioration within 48 hours will be treated as a binary outcome and will be summarised by number (%) of participants with event by treatment group and	Given that the neurological deficit of included subjects was relatively serious, we suggested that modified Rankin Scale scoring 0-1 at 90 days was more appropriate as secondary

binary outcomes)	analysed in a similar way as the primary endpoint by means of binary logistic regression.	analysed in a similar way as the primary endpoint by means of binary logistic regression.	outcome than modified Rankin Scale scoring 0-2 at 90 days.

Supplementary Note 7: Related materials

I: CONSORT CHECKLIST FOR ABSTRACT

Item	Description	Reported on line number
Title	Identification of the study as randomized	Page 1
Authors *	Contact details for the corresponding author	Page 1
Trial design	Description of the trial design (e.g. parallel, cluster, non-inferiority)	Line 4
Methods		
Participants	Eligibility criteria for participants and the settings where the data were collected	Line 6
Interventions	Interventions intended for each group	Line 7
Objective	Specific objective or hypothesis	Line 2-4
Outcome	Clearly defined primary outcome for this report	Line 8-10
Randomization	How participants were allocated to interventions	Line 7
Blinding (masking)	Whether or not participants, care givers, and those assessing the outcomes were blinded to group assignment	Line 10-11
Results		
Numbers randomized	Number of participants randomized to each group	Line 11
Recruitment	Trial status	Line 6
Numbers analysed	Number of participants analysed in each group	Line 12-13
Outcome	For the primary outcome, a result for each group and the estimated effect size and its precision	Line 13-15
Harms	Important adverse events or side effects	Line 16
Conclusions	General interpretation of the results	Line 17-19
Trial registration	Registration number and name of trial register	Line 19
Funding	Source of funding	NA

II: CONSORT CHECKLIST FOR HARMS

Table 2. **Checklist of Items To Include When Reporting Harms in Randomized, Controlled Trials***

Standard CONSORT Checklist: Paper Section and Topic	Standard CONSORT Checklist: Item Number	Descriptor	Reported on Page Number
Title and abstract	1	If the study collected data on harms and benefits, the title or abstract should so state.	1, 3
Introduction			
Background	2	If the trial addresses both harms and benefits, the introduction should so state.	4
Methods			
Participants	3		
Interventions	4		
Objectives	5		
Outcomes	6	List addressed adverse events with definitions for each (with attention, when relevant, to grading, expected vs. unexpected events, reference to standardized and validated definitions, and description of new definitions). Clarify how harms-related information was collected (mode of data collection, timing, attribution methods, intensity of ascertainment, and harms-related monitoring and stopping rules, if pertinent).	7
Sample size	7		
Randomization			
Sequence generation	8		
Allocation concealment	9		8
Implementation	10		
Blinding (masking)	11		
Statistical methods	12	Describe plans for presenting and analyzing information on harms (including coding, handling of recurrent events, specification of timing issues, handling of continuous measures, and any statistical analyses).	
Results			
Participant flow	13	Describe for each arm the participant withdrawals that are due to harms and their experiences with the allocated treatment.	8
Recruitment	14		
Baseline data	15		
Numbers analyzed	16	Provide the denominators for analyses on harms.	
Outcomes and estimation	17	Present the absolute risk per arm and per adverse event type, grade, and seriousness, and present appropriate metrics for recurrent events, continuous variables, and scale variables, whenever pertinent.†	9
Ancillary analyses	18	Describe any subgroup analyses and exploratory analyses for harms.†	
Adverse events	19		
Discussion			
Interpretation	20	Provide a balanced discussion of benefits and harms with emphasis on study limitations, generalizability, and other sources of information on harms.‡	11
Generalizability	21		
Overall evidence	22		

* This proposed extension for harms includes 10 recommendations that correspond to the original CONSORT checklist.
† Descriptors refer to items 17, 18, and 19.
‡ Descriptor refers to items 20, 21, and 22.

Copyright ©2015 American College of Physicians

III: COMMITTEE MEMBERS

Steering Committee

- Xun-Ming Ji (Chair, Xuanwu Hospital, Capital Medical University, Beijing, China)
- Hui-Sheng Chen (Chief Investigator, General Hospital of Northern Theater Command, Shenyang, China)
- Cheng-Shu Zhou (Co-Principal Investigator, Anshan Changda Hospital, Anshan, China)
- Yu-Tong Ma (Co-Principal Investigator, Beipiao Central Hospital, Beipiao, China)
- Hong Zhang (Co-Principal Investigator, Fukuang General Hospital of Liaoning Health Industry Group, Fushun, China)
- Chang-Hao Jiang (Co-Principal Investigator, The Traditional Medicine Hospital of Dalian Lvshunkou, Dalian, China)
- Zhen Jiao (Co-Principal Investigator, Anshan Central Hospital, Anshan, China)
- Run-Hui Li (Co-Principal Investigator, The Affiliated Central Hospital of Shenyang Medical College, Shenyang, China)
- Li-Shu Wan (Co-Principal Investigator, Dandong First Hospital, Dandong, China)
- Zhuo Li (Co-Principal Investigator, Panjin Central Hospital, Panjin, China)
- Ting-Guang Yan (Co-Principal Investigator, Chaoyang Central Hospital, Chaoyang, China)
- Duo-Lao Wang (Senior Medical Statistician, Liverpool School of Tropical Medicine, Liverpool, UK)
- Xin-Hong Wang (Senior Trials Manager, General Hospital of Northern Theater Command, Shenyang, China)
- Xiao-Qiu Li (Senior Trials Manager, General Hospital of Northern Theater Command, Shenyang, China)
- Dan Wang (Trials Manager, General Hospital of Northern Theater Command, Shenyang, China)
- Yu Cui (Medical Statistician, Shenyang Pharmaceutical University, Shenyang, China)

Data Monitoring Committee

- Yi-Long Wang (Chair, Tiantan Hospital, Capital Medical University, Beijing, China)
- Xiao-Wen Hou (Epidemiology, Shenyang Medical College, Shenyang, China)
- Yi Yang (Neurology, First Hospital of Jilin University, Changchun, China)
- Yu-Song Pan (Medical Statistics, Tiantan Hospital, Capital Medical University, Beijing, China)

Institution Human Research Ethics Committee

- Bao-Jun Liu (Chair, General Hospital of Northern Theater Command, Shenyang, China)
- Ping Chen (Associate-chair, General Hospital of Northern Theater Command, Shenyang, China)
- Xiao-Zhong Guo (General Hospital of Northern Theater Command, Shenyang, China)
- Long Liu (General Hospital of Northern Theater Command, Shenyang, China)
- Xiao-Zeng Wang (General Hospital of Northern Theater Command, Shenyang, China)
- Zhen-Dong Zheng (General Hospital of Northern Theater Command, Shenyang, China)
- Rong-Wu Xiang (Shenyang Pharmaceutical University, Shenyang, China)
- Dong Jiang (Liaoning Hehao Law Office, Shenyang, China)
- Bin Lin (Shenyang Sport University, Shenyang, China)

HOPES2 Staff and Support

- Qiu-Shuang Wang (Cerebrovascular Disease Collaboration Innovation Alliance, Shenyang, China)
- Meng-Yao Ge (Cerebrovascular Disease Collaboration Innovation Alliance, Shenyang, China)
- Lin Tao (General Hospital of Northern Theater Command, Shenyang, China)

IV: LIST OF INVESTIGATORS IN HOPES2 TRIAL

* indicates principal investigator in the center.

† indicates blinded assessors in the outcome assessment.

Department of Neurology, General Hospital of Northern Theater Command, Shenyang, China

- Hui-Sheng Chen*
- Xin-Hong Wang
- Dan Gao
- Jin-Di Yang
- Shuang Bao
- Yu-Hang Dong
- Ze-Yang Yu
- Xian-Hui Sun
- Ji-Jie Wang
- Yan-Ni Du
- Yi-Han Wang
- Xiao-Long Li
- Yue Cao
- Wei-Jun Hao
- Zi-Ai Zhao
- Hai-Ying Wu
- Yue Wang
- Xiao-Fu Tian
- Lu Wang†

Department of Neurology, Anshan Changda Hospital, Anshan, China

- Cheng-Wu Zhou*
- Gui-Ying Guo
- Dong-Hui Li
- Qing-Wei Zhao
- Yu-Hua Wang
- Guo-Hua Yu
- Fan Zhang
- Ming-Yan Yu
- Qiang Xing
- Jia-Wei Shi
- Xu-Huan Li
- Jiu-Zhi Li
- Jing-Bo Li
- Hui-Ping Zhang†

Department of Neurology, Beipiao Central Hospital, Beipiao, China

- Yu-Tong Ma*
- Wei Tang
- Ran Zhu
- Nan Jiang
- Li-Gang Feng
- Zheng-Kui Wang

- Fei Jiang†

Department of Neurology, Fukuang General Hospital of Liaoning Health Industry Group, Fushun, China

- Hong Zhang*
- Yan-Zhang Xiao
- Qi Bai†

Department of Neurology, The Traditional Medicine Hospital of Dalian Lvshunkou, Dalian, China

- Chang-Hao Jiang*
- Qi Zhao
- Cheng Fang
- Shan-Hao Zhao
- Peng Wang
- Yang Sun
- Yue Pan†

Department of Neurology, Anshan Central Hospital, Anshan, China

- Zhen Jiao*
- Yu Zhang
- Shi-Hui Li
- Xue Wang
- Xin Wang
- Dong-Mei Zhang†

Department of Neurology, The Affiliated Central Hospital of Shenyang Medical College, Shenyang, China

- Run-Hui Li *
- Tian-Ming Cao
- Han-Shu Li†

Department of Neurology, Dandong First Hospital, Dandong, China

- Li-Shu Wan*
- Xia Xu
- Zhao-Cheng Gong†

Department of Neurology, Panjin Central Hospital, Panjin, China

- Zhuo Li *
- Ying-Li Zhang
- Jia-Jun Liu†

Department of Neurology, Chaoyang Central Hospital, Chaoyang, China

- Ting-Guang Yan*
- Xiao-Qian Jin
- Gang Liu†

V: RECRUITMENT BY SITE IN HOPES2 TRIAL

Inclusion site	Number of patients recruited
Department of Neurology, General Hospital of Northern Theater Command	32
Department of Neurology, Anshan Changda Hospital	21
Department of Neurology, Beipiao Central Hospital	13
Department of Neurology, The Traditional Medicine Hospital of Dalian Lvshunkou	7
Department of Neurology, Fukuang General Hospital of Liaoning Health Industry Group	7
Department of Neurology, Anshan Central Hospital	6
Department of Neurology, The Affiliated Central Hospital of Shenyang Medical College	3
Department of Neurology, Dandong First Hospital	3
Department of Neurology, Panjin Central Hospital	2
Chaoyang Central Hospital	2
