# Supplementary material for: A randomized trial of Trendelenburg position for acute moderate ischemic stroke
Source: Nat Commun. 2023 May 5;14:2592. doi: 10.1038/s41467-023-38313-y (PMC10163013; doi:10.1038/s41467-023-38313-y)
Supplement: Supplementary file 3 — Reporting Summary [file 41467_2023_38313_MOESM3_ESM.pdf]

nature portfolio

Corresponding author(s): Hui-Sheng Chen

Last updated by author(s): Mar 20, 2023

Reporting Summary

Nature Portfolio wishes to improve the reproducibility of the work that we publish. This form provides structure for consistency and transparency in reporting. For further information on Nature Portfolio policies, see our Editorial Policies and the Editorial Policy Checklist.

Statistics

For all statistical analyses, confirm that the following items are present in the figure legend, table legend, main text, or Methods section.

n/a	Confirmed	
☐	☒	The exact sample size (*n*) for each experimental group/condition, given as a discrete number and unit of measurement
☒	☐	A statement on whether measurements were taken from distinct samples or whether the same sample was measured repeatedly
☐	☒	The statistical test(s) used AND whether they are one- or two-sided<br>*Only common tests should be described solely by name; describe more complex techniques in the Methods section.*
☐	☒	A description of all covariates tested
☐	☒	A description of any assumptions or corrections, such as tests of normality and adjustment for multiple comparisons
☐	☒	A full description of the statistical parameters including central tendency (e.g. means) or other basic estimates (e.g. regression coefficient) AND variation (e.g. standard deviation) or associated estimates of uncertainty (e.g. confidence intervals)
☐	☒	For null hypothesis testing, the test statistic (e.g. *F*, *t*, *r*) with confidence intervals, effect sizes, degrees of freedom and *P* value noted<br>*Give P values as exact values whenever suitable.*
☒	☐	For Bayesian analysis, information on the choice of priors and Markov chain Monte Carlo settings
☒	☐	For hierarchical and complex designs, identification of the appropriate level for tests and full reporting of outcomes
☒	☐	Estimates of effect sizes (e.g. Cohen's *d*, Pearson's *r*), indicating how they were calculated

Our web collection on statistics for biologists contains articles on many of the points above.

Software and code

Policy information about availability of computer code

Data collection	no software was used for data collection
Data analysis	IBM SPSS Statistics 24

For manuscripts utilizing custom algorithms or software that are central to the research but not yet described in published literature, software must be made available to editors and reviewers. We strongly encourage code deposition in a community repository (e.g. GitHub). See the Nature Portfolio guidelines for submitting code & software for further information.

Data

Policy information about availability of data

All manuscripts must include a data availability statement. This statement should provide the following information, where applicable:

- Accession codes, unique identifiers, or web links for publicly available datasets
- A description of any restrictions on data availability
- For clinical datasets or third party data, please ensure that the statement adheres to our policy

De-identified data collected for the study including age, sex, baseline NIHSS score, treatment allocation, and functional outcome will be shared 2 years after publication by requesting the corresponding author (Hui-Sheng Chen, email: chszh@aliyun.com) for academic purposes. The corresponding author will reply to the request within 2 months, subject to approval of the ethics committees of the General Hospital of Northern Theatre Command.

Human research participants

Policy information about studies involving human research participants and Sex and Gender in Research.

Reporting on sex and gender	we used the "sex" term. The findings of current study does not apply only one sex. sex was considered in the study design and was randomly assigned into experimental or control group. we did not collected the data disaggregated sex. sex-based analysis was not performed.
Population characteristics	Eligible patients were adults and the median age was 62.6 (32, 86) years, including 68 (72.3%) males and 26 (27.7) females. past and current diagnosis includes the current diagnosis including hypertension 56 (59.6%), diabetes 26 (27.7%), Coronary heart disease 10 (10.6%) and previous stroke 32 (34.0%). Treatment categories were divided into two group, patients in the HDP group receiving -20° Trendelenburg, and the control group were treated according to the AHA/ASA 2018 guidelines for early management of ischemic stroke.
Recruitment	Eligible patients were adults aged 18 years or older with acute moderate ischemic stroke (defined as baseline NIHSS scores 6 to 16) with probable LAA etiology (based on the head and neck CTA or MRA imaging) at the time of randomization who had been functioning independently in the community (mRS scores 0 to 1) before the stroke, and were enrolled up to 24 hours after onset of stroke symptoms. Key exclusion criteria were that a patient received intravenous thrombolysis and/or endovascular therapy, and planned carotid or intracranial revascularization within 90 days, other etiologies, any possible contraindication to head-down position (e.g., active vomiting, pneumonia, uncontrolled heart failure, and need for enteral feedings). The bias present below: first, the relatively small sample make the conclusion exploratory and subgroup analysis such as effect of site on primary outcome impossible. Second, the open-label design may have resulted in bias, although we used blinded evaluation at 90 days to mitigate this potential bias. Third, the highly selected population, for example excluding patients who received thrombolysis or thrombectomy, limited to anterior circulation stroke, introduce selection bias and may limit generalizability of our results.
Ethics oversight	The General Hospital of Northern Theatre Command, Anshan Changda Hospital, Beipiao Central Hospital, Fukuang General Hospital of Liaoning Health Industry Group, The Traditional Medicine Hospital of Dalian Lvshunkou, Central Hospital affiliated to Shenyang Medical College, Dandong First Hospital, Anshan Central Hospital; Panjin Central Hospital, and Chaoyang Central Hospital.

Note that full information on the approval of the study protocol must also be provided in the manuscript.

Field-specific reporting

Please select the one below that is the best fit for your research. If you are not sure, read the appropriate sections before making your selection.

☒ Life sciences ☐ Behavioural & social sciences ☐ Ecological, evolutionary & environmental sciences

For a reference copy of the document with all sections, see nature.com/documents/nr-reporting-summary-flat.pdf

Life sciences study design

All studies must disclose on these points even when the disclosure is negative.

Sample size	No formal sample size calculation was performed due to no relevant data availability from previous trials. For this exploratory trial, the sample size (50 patients per group) was based on the recommendation of the Steering Committee.
Data exclusions	In this trial, 96 eligible patients were randomly assigned to the HDP group (n=47) and control group (n=49). After 2 patients were excluded, 94 patients were included in the mITT population (46 in the HDP group and 48 in the control group). The procedure was completed according to the protocol for 89 patients (42 in the HDP group and 47 in the control group), which was included in the per-protocol analysis. There were no cross-overs between groups in the trial.
Replication	To verify the reproducibility of the experimental findings, we did a sensitivity analysis. The primary outcomes were adjusted for confounding covariates (age, NIHSS score at randomization, the degree of related vessel stenosis, onset to randomization time, and location of responsible vessels). And missing values in the primary outcome were imputed using the last observation carried forward method, the worst-case scenario, best-case scenario approaches.
Randomization	In this trial, eligible patients were randomly assigned (1:1) using a computer-generated randomization sequence with block size of four and sealed envelopes, prepared by an independent statistician, into either HDP group receiving Trendelenburg as an adjunct to guideline-based medical management, or control group only receiving guideline-based medical management.
Blinding	The treatment was not blinded, but final 90-day mRS was evaluated by one qualified personnel who was blinded to treatment allocation

Reporting for specific materials, systems and methods

We require information from authors about some types of materials, experimental systems and methods used in many studies. Here, indicate whether each material, system or method listed is relevant to your study. If you are not sure if a list item applies to your research, read the appropriate section before selecting a response.

Materials & experimental systems

n/a	Involved in the study
☒	☐ Antibodies
☒	☐ Eukaryotic cell lines
☒	☐ Palaeontology and archaeology
☒	☐ Animals and other organisms
☐	☒ Clinical data
☒	☐ Dual use research of concern

Methods

n/a	Involved in the study
☒	☐ ChIP-seq
☒	☐ Flow cytometry
☒	☐ MRI-based neuroimaging

Clinical data

Policy information about clinical studies

All manuscripts should comply with the ICMJE guidelines for publication of clinical research and a completed CONSORT checklist must be included with all submissions.

Clinical trial registration: This trial was registered with ClinicalTrials.gov with number NCT03744533

Study protocol: please see study protocol in Supplementary Note 1-3 in Supplementary information

Data collection: The data was collected by investigators at each center. Demographic and clinical details were obtained at randomization. Follow-up data were collected at 7 days, 12 days (or at hospital discharge if earlier), and 90 days after randomization. Final 90-day mRS was evaluated by one qualified personnel who was blinded to treatment allocation according to a standardized procedure manual in each study center. The data collected between Nov 16, 2018, and Aug 28, 2021.

Outcomes: The primary endpoint was the proportion of favorable functional outcome defined as 90-day mRS score of 0-2. The mRS ranging from 0 (no symptoms) to 6 (death). Secondary outcomes included mRS score 0-1 at 90 days, early neurological deterioration (defined as ≥ 4 increase in NIHSS score within 48 hours, but not result of cerebral haemorrhage), change in NIHSS score at day 12 compared with baseline(NIHSS scores range from 0 to 42, with higher scores indicating greater stroke severity), occurrence of stroke or other vascular events, and death due to any cause within 90 days. NIHSS was assessed by local investigator who was not blinded to treatment allocation. Final 90-day mRS was evaluated by one qualified personnel who was blinded to treatment allocation according to a standardized procedure manual in each study center.
